# Genome-wide analysis of cotton GH3 subfamily II reveals functional divergence in fiber development, hormone response and plant architecture

**DOI:** 10.1186/s12870-018-1545-5

**Published:** 2018-12-12

**Authors:** Daoqian Yu, Ghulam Qanmber, Lili Lu, Lingling Wang, Jie Li, Zhaoen Yang, Zhao Liu, Yi Li, Quanjia Chen, Venugopal Mendu, Fuguang Li, Zuoren Yang

**Affiliations:** 1 Xinjiang Research Base, State Key Laboratory of Cotton Biology, Xinjiang Agricultural University, Urumqi, 830052 China; 2 State Key Laboratory of Cotton Biology, Key Laboratory of Biological and Genetic Breeding of Cotton, Institute of Cotton Research, Chinese Academy of Agricultural Sciences, Anyang, 455000 China; 3 Zhengzhou Research Base, State Key Laboratory of Cotton Biology, Zhengzhou University, Zhengzhou, 450000 China; 4 Fiber and Biopolymer Research Institute (FBRI), Department of Plant and Soil Science, Texas Tech University, Lubbock, TX 79409 USA

**Keywords:** *Gossypium hirsutum*, Gene family, GH3, Phytohormone, Expression patterns, *cis*-regulatory element

## Abstract

**Background:**

Auxin-induced genes regulate many aspects of plant growth and development. The Gretchen Hagen 3 (*GH3*) gene family, one of three major early auxin-responsive families, is ubiquitous in the plant kingdom and its members function as regulators in modulating hormonal homeostasis, and stress adaptations. Specific Auxin-amido synthetase activity of *GH3* subfamily II genes is reported to reversibly inactivate or fully degrade excess auxin through the formation of amino acid conjugates. Despite these crucial roles, to date, genome-wide analysis of the *GH3* gene family has not been reported in cotton.

**Results:**

We identified a total of 10 *GH3* subfamily II genes in *G. arboreum*, 10 in *G. raimondii*, and 20 in *G. hirsutum*, respectively. Bioinformatic analysis showed that cotton *GH3* genes are conserved with the established *GH3s* in plants. Expression pattern analysis based on RNA-seq data and qRT-PCR revealed that 20 *GhGH3* genes were differentially expressed in a temporally and spatially specific manner, indicating their diverse functions in growth and development. We further summarized the organization of promoter regulatory elements and monitored their responsiveness to treatment with IAA (indole-3-acetic acid), SA (salicylic acid), GA (gibberellic acid) and BL (brassinolide) by qRT-PCR in roots and stems. These hormones seemed to regulate the expression of *GH3* genes in both a positive and a negative manner while certain members likely have higher sensitivity to all four hormones. Further, we tested the expression of *GhGH3* genes in the BR-deficient mutant *pag1* and the corresponding wild-type (WT) of CCRI24. The altered expression reflected the true responsiveness to BL and further suggested possible reasons, at least in part, responsible for the dramatic dwarf and shriveled phenotypes of *pag1*.

**Conclusion:**

We comprehensively identified *GH3* subfamily II genes in cotton. *GhGH3s* are differentially expressed in various tissues/organs/stages. Their response to IAA, SA, BL and GA and altered expression in *pag1* suggest that some *GhGH3* genes might be simultaneously involved in multiple hormone signaling pathways. Taken together, our results suggest that members of the *GhGH3* gene family could be possible candidate genes for mechanistic study and applications in cotton fiber development in addition to the reconstruction of plant architecture.

**Electronic supplementary material:**

The online version of this article (10.1186/s12870-018-1545-5) contains supplementary material, which is available to authorized users.

## Background

As the first class of plant growth-promoting substances discovered more than 70 years ago, auxin is a critical plant hormone, playing pivotal roles in root and shoot architecture, vascular development, organ patterning, reproduction, hormonal crosstalk and responses to light, gravity and biotic and abiotic stimuli [1–4]. The effect of auxin on plant growth and development is achieved by regulating the expression of downstream target genes of auxin signaling. Among them, three major groups, auxin/indole-3-acetic acid (*Aux/IAA*), small auxin up RNA (*SAUR*) and Gretchen Hagen 3 (*GH3*) have been designated as early auxin-responsive gene families characterized by rapid expression changes on exposure to exogenous auxin [5–7]. Genome-wide isolation and characterization of early auxin-responsive family genes from many plant species has provided an opportunity for better understanding of the molecular mechanisms of auxin action [7–9].

The GH3 family of proteins were found to be ubiquitously present in numerous plant species and some vertebrates such as mouse, but not in the sequenced genomes of yeast, *Caenorhabditis elegans* or Drosophila [7, 10]. In 1984, the first *GH3* gene was isolated from soybean by differential hybridization screening as an auxin-induced cDNA clone from etiolated hypocotyls [11]. *GH3* family genes were first comprehensively identified in the model plant Arabidopsis [7]. To date, genome-wide analysis of the *GH3* gene family has been completed in rice [8, 12], grape [13], rosids [14], tomato [15], legumes [10], *Medicago truncatula* [16], apple [17], and maize [4]. In general, the chromosomal distribution of *GH3* genes is uneven and some members tend to be clustered [7, 16]. A majority of *GH3* genes encode proteins of about 70 kDa and comprise 3–5 exons separated by introns of different lengths [6]. The promoter region of most *GH3* genes contains a common *cis*-element (AuxREs, TGTCTC) recognized by auxin-response factors (ARFs). When treated with active auxin, the transcript level rapidly increases within 0.25–0.5 h and peaks a maximum of 2–4 h after treatment [11]. In addition to the specific response to auxin, *GH3* genes are also transcriptionally regulated by stress-related hormones ABA (abscisic acid), JA (jasmonic acid) and SA (salicylic acid); growth-promoting hormones GA (gibberellins) and BRs (brassinosteroids); ripening/senescence-associated hormone ETH (ethylene); biotic stresses of pathogens; and abiotic stresses of light, salt, drought, cold, and so on [10, 16]. Moreover, *GH3* genes are transcribed in a spatial- and temporal-specific manner, and the cytoplasmic-localized GH3 protein appears to be more abundant and stable than Aux/lAA and SAUR proteins [18]. Previous studies have shown that GH3 proteins maintain dynamic homeostasis or activity of IAA (indole-3-acetic acid), SA, and JA through the formation of hormone-amino acid conjugates [19, 20]. Notably, the fates of hormone conjugates seem to be diverse; formation of a JA-Ile conjugate can activate JA and enhance some jasmonate responses while several other forms of JA conjugates appear to be inactive [21]. The action of IAA-amido synthetases is more related to reducing the amount of excess active IAA by degradation (IAA-Asp/Glu) or temporary storage (IAA-Ala/Leu) [19]. Although some *GH3* genes have been shown to form SA conjugates, their biochemical function remains unclear [20, 22–26].

Based on sequence similarity and substrate specificities, a total of 20 *GH3* family genes in Arabidopsis have been classified into three subfamilies (I-III) [19]. In particular, 10 enzymes of subfamily III (*AtGH3.7*–*3.8*, *AtGH3.12*–*3.16* and *AtGH3.18*–*3.20*) seem to be species specific and have only been identified in Arabidopsis to date, although some of these do not appear to be active in response to IAA or other plant hormones [20]. AtGH3.12/PBS3 is the only characterized member of subfamily III that is involved in SA metabolism, and is induced in response to avirulent or virulent *Pseudomonas syringae* pathogen infection [22, 23]. Subfamily I consists of *AtGH3.10* and *AtGH3.11/JAR1/FIN219*, and the latter is the only enzyme functionally documented in subgroup I to activate JA by forming JA-amino acid conjugates [21]. The remaining members, *AtGH3.1*–*3.6*, *AtGH3.9* and *AtGH3.17*, belong to subgroup II. All Arabidopsis subgroup II enzymes but AtGH3.1, which was not analyzed, were demonstrated to be IAA responsive [19]. It was noted that the IAA-amido synthetases are restricted to the eight enzymes of subfamily II in Arabidopsis, and the same may be true for GH3 enzymes of subfamily II from other plant species [19]. The subfamily II enzymes are involved in auxin homeostasis, and when the normal level of *GH3* genes is disrupted, distinct but interrelated phenotypes can be observed [27]. The gain-of-function mutant *ydk1-D*, generated by an activation-tagged insertion proximal to the *GH3.2* gene in Arabidopsis, shows a short primary root, reduced lateral root number, reduced apical dominance and short hypocotyl both in light and dark [28]. *Wes1-D* and *gh3.5-1D*, both resulting from activation of the *GH3.5* gene by insertion of the 35S enhancer, exhibit auxin-deficient traits. The adult plant exhibits exaggerated dwarfism with small curly rosette leaves, short primary roots and a reduced number of lateral roots [24, 25]. With the exception of almost unchanged primary root length, a similar phenotype is found in the Arabidopsis *dfl1-D* mutant, in which the *GH3.6* gene is over-expressed following a T-DNA insertion [29]. *OsGH3.13/TLD1* is the rice ortholog of Arabidopsis *GH3.17*, and its activation results in alterations in plant architecture and tissue patterning, displaying a typical auxin-deficient phenotype of dwarfism, increased tillers, enlarged leaf angles, and improved drought tolerance [30].

Cotton (*Gossypium* spp.) is an important crop plant. The widely cultivated *G. hirsutum* (AD1-genome) is an allotetraploid originating from two diploid ancestor species, *G. arboreum* (A-genome) and *G. raimondii* (D-genome), through a natural hybridization and genome doubling process 1–2 MYA (Million Years Ago) [31, 32]. Cotton fiber, the most prevalent natural fiber used in the textile industry, is a single-celled seed hair derived from seed coat epidermal cells. The elongated fiber cells, composed of nearly pure cellulose, can reach lengths of nearly 6 cm [33]. With several commercially important products, cotton represents an important model plant and commercial crop for the study of polyploidy, cell elongation and cell wall synthesis [34]. Various studies on cotton fiber cell development have implicated auxin as a critical regulator of fiber development. IAA is an important component for fiber growth in in vitro-cultured cotton ovules [33]. Targeted expression of the IAA biosynthetic gene *iaaM* driven by the promoter of the *FBP7* gene increased IAA levels in the epidermis of cotton ovules at the fiber initiation stage and substantially increased the number of lint fibers [35]. In addition, reconstruction of ideal plant architecture is imperative for mechanization in cotton production. Previous studies reported that altered IAA levels can change plant architecture in Arabidopsis and rice, which presented a new idea for regulating auxin homeostasis in vivo to rebuild cotton plant architecture [24, 28–30, 36]. The main function of *GH3* family genes is to maintain dynamic homeostasis of active IAA. However, no genome-wide analysis of *GH3* family genes in cotton has been published to date, especially of subfamily II GH3s with IAA-amino synthetase activity. The availability of genome sequences and the established *GH3* gene family of other plant species provides opportunities for comprehensive analysis of the *GH3* gene family in cotton.

In this study, we performed genome-wide identification and expression analysis of subfamily II GH3s in tetraploid *G. hirsutum* and two diploid species *G. arboreum* and *G. raimondii*. A phylogenetic tree was built to analyze the evolutionary relationships of GH3s between cotton and other plant species. In particular, the gene structure, conserved motif, expression pattern in root, stem, leaf, flower, fiber and ovule in different development stages, *cis*-regulatory elements of promoter sequences, and the responses to BL, GA, IAA and SA were examined systematically in *G. hirsutum*. The results of this study provide essential information on GH3 genes with potential application in studies of the mechanism of fiber initiation and development, fiber improvement, and plant architecture modification in cotton.

## Results

### Genome-wide identification and nomenclature of cotton *GH3* subfamily II genes

The Hidden Markov Model (HMM) profile (PF03321.10) was employed to search against protein sequences of three cotton species (see below) [37–39]. In the first round of screening, using a threshold value of 10^− 5^, 49, 39, and 56 tentative genes of the GH3 family were obtained in *G. arboreum* (BGI, v2.0), *G. raimondii* (BGI, v1.0), and *G. hirsutum* (NAU, v1.1), respectively (Additional file 1: Table S1, sheet1–3). To identify the cotton orthologs of Arabidopsis GH3s, each set of tentative cotton GH3s was aligned with 20 AtGH3 proteins using ClustalX 1.83, and three neighbor-joining (NJ) phylogenetic trees were then constructed independently using PHYLIP 3.6 or MEGA7 based on the sequence alignment (Additional file 2: Figure S1 a, b and c). This strategy resulted in the identification of 17, 16, and 34 candidate genes of the GH3 family in *G. arboreum*, *G. raimondii*, and *G. hirsutum*, respectively (Additional file 1: Table S1, sheet1–3). Despite the presence of a putative GH3 domain, members clustered into *AtGH3.20* in *G. arboreum* and *G. raimondii* were disregarded due to the incompleteness of *AtGH3.20* and unexpected clustering compared with that in *G. hirsutum*. We focused on subfamily II *GhGH3s* because of their particular activity as IAA-amido synthetases. Members grouped with subfamily II Arabidopsis *GH3* genes were considered theoretically as subfamily II *GH3* genes in cotton. Conserved domain analysis using the online tool SMART showed the presence of a GH3 domain for all candidates. In total, 10, 10, and 20 candidate genes of subfamily II of the *GH3* gene family were identified in *G. arboreum*, *G. raimondii*, and *G. hirsutum*, respectively (Table 1; Additional file 3: Table S2). Most of the *GH3* genes identified here exhibited similar characteristics in ORF (Open Reading Frame) length, molecular weight, intron numbers and isoelectric point, except for *GrGH3.5*, which possessed a 3828 bp ORF according to the annotation of the *G. raimondii* genome (BGI, v1.0). We speculated that the abnormal length of *GrGH3.5* was because of improper genome assembly. To confirm the observation, we used Gh_DGH3.5 from the Dt-subgenome of the *G. hirsutum* genome (NAU, v1.1) as a query to search against the *G. raimondii* genome (JGI, v2.0) and confirmed its homologous gene *GrGH3.5* (locus_ID: Gorai.007G219500.1), which displayed, as expected, a normal ORF length of 1821 bp. We further tested 20 *GhGH3* genes in the latest genome data of *G. hirsutum* (DOE-JGI, v3.1). Except for Gh_DGH3.4 in which two amino acids were deleted at the N-terminus, all GhGH3s shared the same peptide length, intron numbers, and chromosomal distribution (Additional file 4: Table S3). No frame shift mutation was found for any of the *GhGH3* genes. In consideration of the highly similar characteristics of *GH3* genes in the two genome assemblies of *G. hirsutum* (NAU, v1.1 and JGI, v3.1), we selected the widely used annotation of the *G. hirsutum* genome (NAU, v1.1) for subsequent analysis and presentation of subfamily II GH3s in cotton. All the identified proteins were named based on sequence identity with their orthologs in Arabidopsis and their chromosomal locations. The comprehensive information of *GH3* genes in three cotton species, including gene name, locus_IDs, Arabidopsis orthologs, ORF length, intron numbers, chromosomal distribution as well as the length, molecular weight and isoelectric point of deduced polypeptides, are listed in Table 1 and Additional file 3: Table S2.

**Table 1:** Characteristics of subfamily II *GH3s* in *G. hirsutum* (NAU-NBI, v1.1)

Gene name	Locus_ID	Arabidopsis orthologs	ORF	Introns	Chr.	Position				Deduced polypeptide		
						Start	End	Strand		Length (aa)	MW (Da)	pI
Gh_AGH3.1	Gh_A03G1628	AtGH3.1	1797	2	A03	97,446,817	97,448,799	–		598	67,902.66	6.59
Gh_DGH3.1	Gh_D02G2045	AtGH3.1	1797	2	D02	64,687,129	64,689,111	–		598	67,882.61	6.59
Gh_AGH3.2	Gh_A12G0181	AtGH3.1	1794	2	A12	2,676,124	2,678,101	–		597	67,886.41	6.02
Gh_DGH3.2	Gh_D12G0182	AtGH3.1	1797	2	D12	2,358,751	2,360,733	–		598	67,901.46	6.02
Gh_AGH3.3	Gh_A13G0392	AtGH3.1	1809	2	A13	5,213,724	5,216,267	+		602	68,094.70	5.55
Gh_DGH3.3	Gh_D13G0434	AtGH3.1	1809	2	D13	4,919,812	4,922,365	+		602	68,036.63	5.59
Gh_AGH3.4	Gh_A11G0443	AtGH3.1	1779	2	A11	4,184,163	4,186,123	+		592	67,383.32	5.58
Gh_DGH3.4	Gh_D11G0514	AtGH3.1	1779	2	D11	4,476,391	4,478,353	+		592	67,370.40	5.83
Gh_AGH3.5	Gh_A11G1993	AtGH3.1	1821	2	A11	56,447,917	56,449,936	–		606	68,573.57	5.60
Gh_DGH3.5	Gh_D11G1989	AtGH3.1	1821	2	D11	26,018,863	26,020,882	+		606	68,492.53	5.53
Gh_AGH3.6	Gh_A01G0546	AtGH3.5/3.6	1842	2	A01	9,238,374	9,240,450	+		613	69,496.44	5.91
Gh_DGH3.6	Gh_D01G0557	AtGH3.5/3.6	1842	2	D01	7,266,590	7,268,663	+		613	69,476.51	5.87
Gh_AGH3.7	Gh_A01G0547	AtGH3.5/3.6	1842	2	A01	9,407,662	9,409,824	+		613	69,261.22	6.08
Gh_DGH3.7	Gh_D01G0559	AtGH3.5/3.6	1842	2	D01	7,427,672	7,429,831	+		613	69,183.12	6.08
Gh_AGH3.8	Gh_A03G1429	AtGH3.5/3.6	1848	2	A03	94,060,935	94,062,983	+		615	69,133.16	5.51
Gh_AGH3.9	Gh_A11G1054	AtGH3.9	1788	3	A11	12,002,212	12,005,380	+		595	67,035.10	5.58
Gh_DGH3.9	Gh_D11G1209	AtGH3.9	1788	3	D11	11,289,480	11,292,643	+		595	66,823.59	5.41
Gh_AGH3.17	Gh_A03G1354	AtGH3.17	1935	4	A03	92,377,252	92,379,703	–		644	73,210.99	6.18
Gh_DGH3.17	Gh_D02G1794	AtGH3.17	1935	4	D02	60,662,189	60,664,648	–		644	73,258.06	6.81
Gh_AGH3.18	Gh_A04G0874	AtGH3.17	1629	4	A04	56,316,128	56,318,166	–		542	61,206.77	5.97

Characteristics of *GH3s* in latest genomes of *G. hirsutum* (DOE-JGI, v3.1) is shown in Additional file 4 Table S3

### Systematic bioinformatic analysis of cotton *GH3* subfamily II genes

**Fig. 1:**
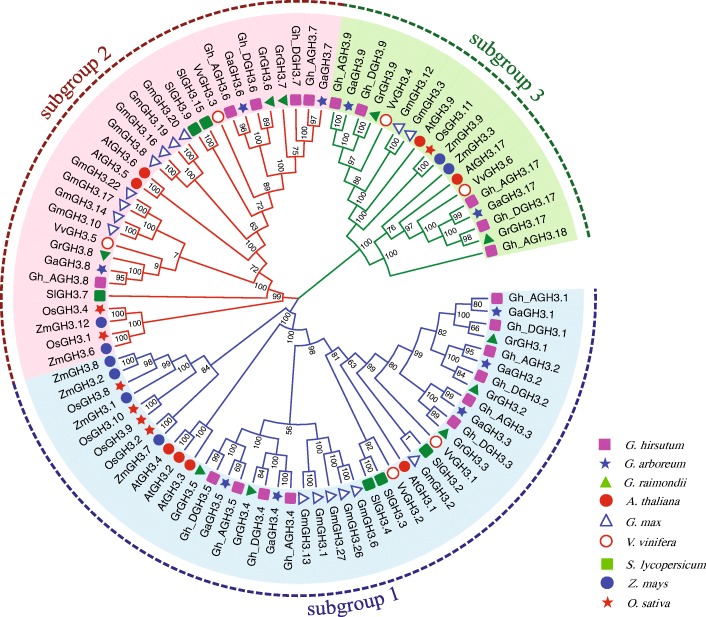
Phylogenetic relationships of subfamily II GH3s in plants. GH3s identified in three cotton species and established subfamily II GH3s from *A .thaliana, S. lycopersicum, G. max, O. sativa, Z.mays* and *V. vinifera* were included. Cotton GH3s identified here could be re-classified into subgroup 1–3. GH3s of other plants were unevenly distributed such that no SlGH3 and just one OsGH3 (OsGH3.11) clustered into subgroup 3. Members of VvGH3s, GmGH3s and cotton GH3s always clustered close to each other, exhibiting closer relationships. A neighbor-joining tree was built using MEGA 7.0 and visualized using online tool Evolview. The Gh_A and Gh_D in the upland cotton indicate the At- and Dt-subgenomes, respectively. GH3s belonging to one plant species are marked with the indicated leaf label decorations. Blocks of subgroup 1–3 are highlighted with light blue, pink and light green, respectively. Percentage bootstrap scores were calculated from 1000 iterations

As described above, the *GH3* gene family has been identified in numerous plant species. Various *GH3* genes well-studied in model plants can help us to accurately search for and categorize them and predict their potential roles in cotton. Phylogenetic analysis was performed using neighbor-joining and a phylogenetic tree was constructed with subfamily II *GH3s* from different plant species, including 20 *GhGH3s* (*G. hirsutum*), 10 *GrGH3s* (*G. raimondii*), 10 *GaGH3s* (*G. arboreum*), 8 *AtGH3s* (*A. thaliana*), 6 *SlGH3s* (*S. lycopersicum*), 7 *OsGH3s* (*O. sativa*), 8 *ZmGH3s* (*Z. mays*), 16 *GmGH3s* (*G. max*) and 6 *VvGH3s* (*V. vinifera*). All sequences used in this study are supplied in Additional file 1: Table S1, sheet 4. The resulting tree exhibits clustering in three major groups (designated as subgroups 1–3 to differentiate them from the established subfamilies I-III in Arabidopsis). *Gh_AGH3.1*-*Gh_DGH3.5*, *AtGH3.1*-*AtGH3.4* together with two *VvGH3s*, three *SlGH3s*, four *ZmGH3s*, four *OsGH3s,* and six *GmGH3s* fell into subgroup 1 while *Gh_AGH3.9*-*Gh_AGH3.18*, *AtGH3.9*, *AtGH3.17*, *VvGH3.4/3.6*, *OsGH3.11*, *ZmGH3.3/3.9,* and *GmGH3.3/3.12* were allotted to subgroup 3 (Fig. 1). *GH3* genes from nine kinds of plant species were present in almost all three subgroups with the exception that no members of *SlGH3* genes were found in group 3. One out of 7 *OSGH3* genes, *OsGH3.11,* was the only member existed in subgroup 3. Interestingly, *VvGH3s*, *GmGH3s* and cotton *GH3* genes always clustered closely to each other, exhibiting closer relationships compared with other species, which is consistent with the close evolutionary relationships between these three plant species [40].

**Fig. 2:**
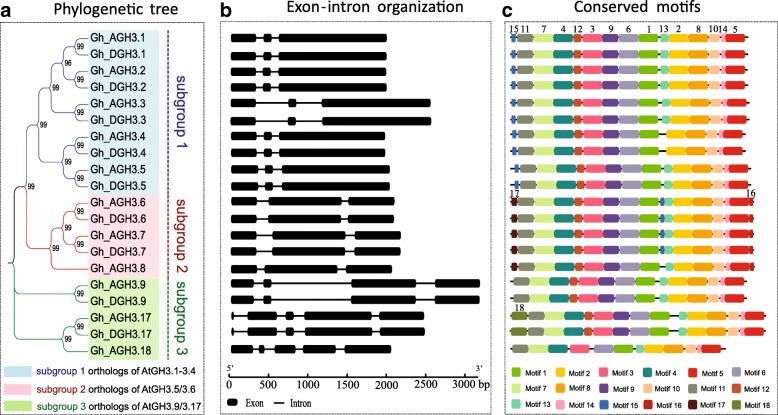
Phylogenetic relationship, gene structure and motif analysis of 20 GhGH3s. **a** The small phylogenetic tree constructed with 20 GhGH3s, clearly exhibiting three subgroups. **b** Exon-intron organizations. Subgroup 1 and 2 have three exons while subgroup 3 genes have four or five. The length of the second exon in subgroup 2 is longer than that of subgroup 1. Black boxes and lines represent exons and introns, respectively. **c** Conserved motif analysis. Except for GhGH3.18, all of the remaining 19 GhGH3s share the same fourteen motifs ranging from 1 to 14. Necessary conserved motifs/residues for hormone-amido synthetase activity were analyzed, and motifs 1, 2 and 4 contain the nucleotide (ATP/AMP)-binding motifs/residues, motifs 3, 4, 6 and 12 possess the hormone-binding motifs/residues. The residues contained in motifs 8, 10 and 12 determine amino-acid preferences. The number above each motif represents its code as described below

**Fig. 3:**
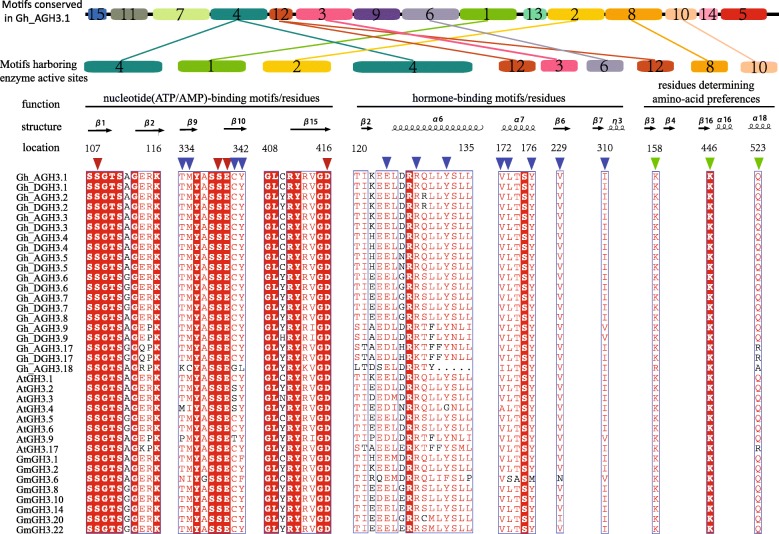
Sequence alignment of conserved residues or motifs. Protein sequences of 20 GhGH3s were aligned using MAFFT with all members of subfamily II in Arabidopsis and some in Soybean. The nucleotide (ATP/AMP) and hormone-binding motifs/residues as well as residues determining amino-acid preferences are conserved, indicating their similar functions as the IAA-amido synthetase in cotton. The colored triangles represent active sites and secondary structures near the active sites are also shown. Numbering at the top indicates the location of residues or motifs corresponding to Gh_AGH3.1. Motifs are put above the corresponding alignment of conserved residues and conserved motifs in full-length Gh_AGH3.1 are also shown as an example to display the locations of all motifs. The same motifs are linked by lines with the same color. The number in each motif indicates its code as described in Fig. 2c. Note that the ends of the lines do not indicate the actual positions of enzyme active sites

As described above, the 20 *GhGH3* genes can be divided into three subgroups. The largest subgroup 1 contained *Gh_AGH3.1*-*Gh_DGH3.5*, showing close relationships to *AtGH3.1*. *Gh_AGH3.6*-*Gh_AGH3.8* belonged to subgroup 2, which seem to be the orthologs of *AtGH3.5/3.6* in cotton. The remaining members constituted subgroup 3 and displayed close relationships to *AtGH3.9* (Fig. 2a). Gene structure analysis suggested that intron numbers were conserved among *GhGH3* genes. Subgroup 1 and 2 possessed three exons while subgroup 3 had four or five. The length of the second exon in subgroup 2 *GH3* genes was markedly longer than that of subgroup 1 (Fig. 2b). Conserved motif analysis showed that all 19 *GhGH3* genes shared the same fourteen motifs except for *GhGH3.18*, among them eight conserved motifs containing known enzyme active sites, with some sites found in the same motifs. For example, motifs 1, 2 and 4 contained nucleotide (ATP/AMP)-binding motifs/residues while motifs 3, 4, 6 and 12 possessed hormone-binding motifs/residues. The residues contained in motifs 8, 10 and 12 determined amino-acid preferences (Fig. 2c; Fig. 3; Additional file 5: Figure S2 and Additional file 6: Figure S3).

**Fig. 4:**
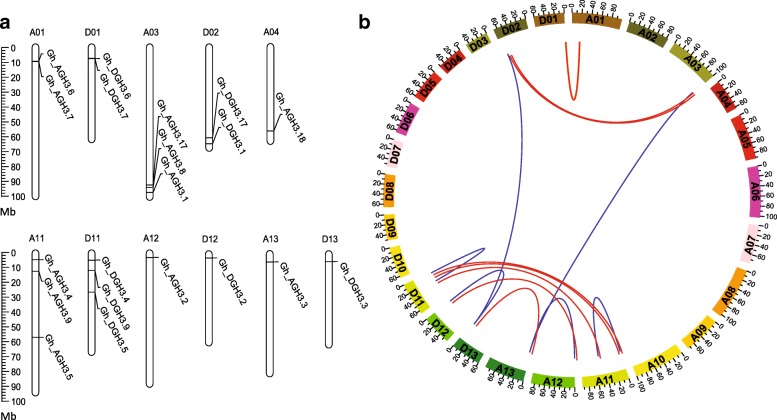
Mapping and synteny analysis of *GhGH3* genes. *GhGH3* genes were named based on sequence identity with their orthologs in Arabidopsis and their chromosomal locations. The capital letters and numbers indicate the At- or Dt-subgenome of *G. hirsutum* and chromosome numbers, respectively. **a** Illustrative diagram of chromosomal distribution of *GhGH3* genes, showing uneven distribution on 11 out of 26 chromosomes. **b** CIRCOS figure of *GhGH3* genes. The orthologous pairs from homologous chromosome pairs, At- and Dt-subgenome of *G. hirsutum,* are linked with red lines while the paralogous genes mapped on non-homologous chromosome pairs are linked with blue lines

Furthermore, we analyzed the chromosomal distribution of *GhGH3* genes. The 20 *GhGH3* genes were unevenly distributed on 11 chromosomes; none were mapped on scaffold fragments (Fig. 4). Chromosomes A03, A11 and D11 each contained three *GhGH3* genes while chromosomes A04, A12, A13, D12 and D13 only had one *GhGH3* gene each. The putative *GhGH3* genes of subfamily I were also mapped to the chromosomes. The largest gene clusters were found on chromosome D11, consisting of *Gh_DGH3.4*, *Gh_DGH3.5*, *Gh_DGH3.9* as well as the unnamed locus_IDs of Gh_D11G1005 and Gh_D11G1006. Additionally, three genes were located on scaffold fragments (Additional file 7: Figure S4). Moreover, synteny analysis displayed the same chromosomal distribution, revealing that most *GH3* loci were highly conserved between At- and Dt-subgenomes. Except for *Gh_AGH3.8* and *Gh_AGH3.18*, all others from the A genome of *G. arboreum* and the D genome of *G. raimondii* were well matched to members of At- and Dt-subgenomes of *G. hirsutum*, forming orthologous pairs.

### Expression pattern analysis of cotton *GH3* genes in vegetative and reproductive tissues/stages

**Fig. 5:**
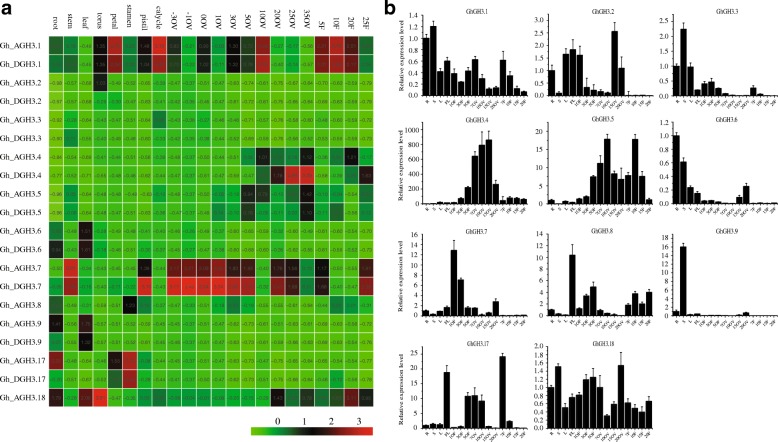
Expression profile of *GhGH3s* based on RNA-seq and qRT-PCR in various tissue/organs/stages. **a** Trends in *GhGH3* gene expression based on publicly available RNA-seq data. Expression of individual genes are shown for 20 *GhGH3s*. Numbers on the x-axis indicate days post anthesis, with negative numbers implying days before anthesis. The prefix OV is for ovules and F for fiber. Green, black and red backgrounds represent low, intermediate and high expression levels, respectively. The original RPKM (reads per kilobase per million) values are normalized to 0–1 and shown in boxes. **b** Pattern of gene expression by qRT-PCR analysis. Expression of allele pairs or individual genes are shown. Values on the y-axis represent relative expression levels while the x-axis indicates days post anthesis. Materials from the field under normal conditions were tagged and sampled. The relative expression levels of *GhGH3* genes were measured in roots (R), stems (S), leaves (L), flowers (FL), early stage developing ovules with attached fibers (1, 3, 5 DPA OF, ovules plus fibers), and different stages of detached ovules and fibers (7, 10, 15, 20 DPA O/F, ovules or fibers). *GhHis* 3 was used as an internal control to normalize the expression data. Error bars show standard deviation calculated from three replicates

**Fig. 6:**
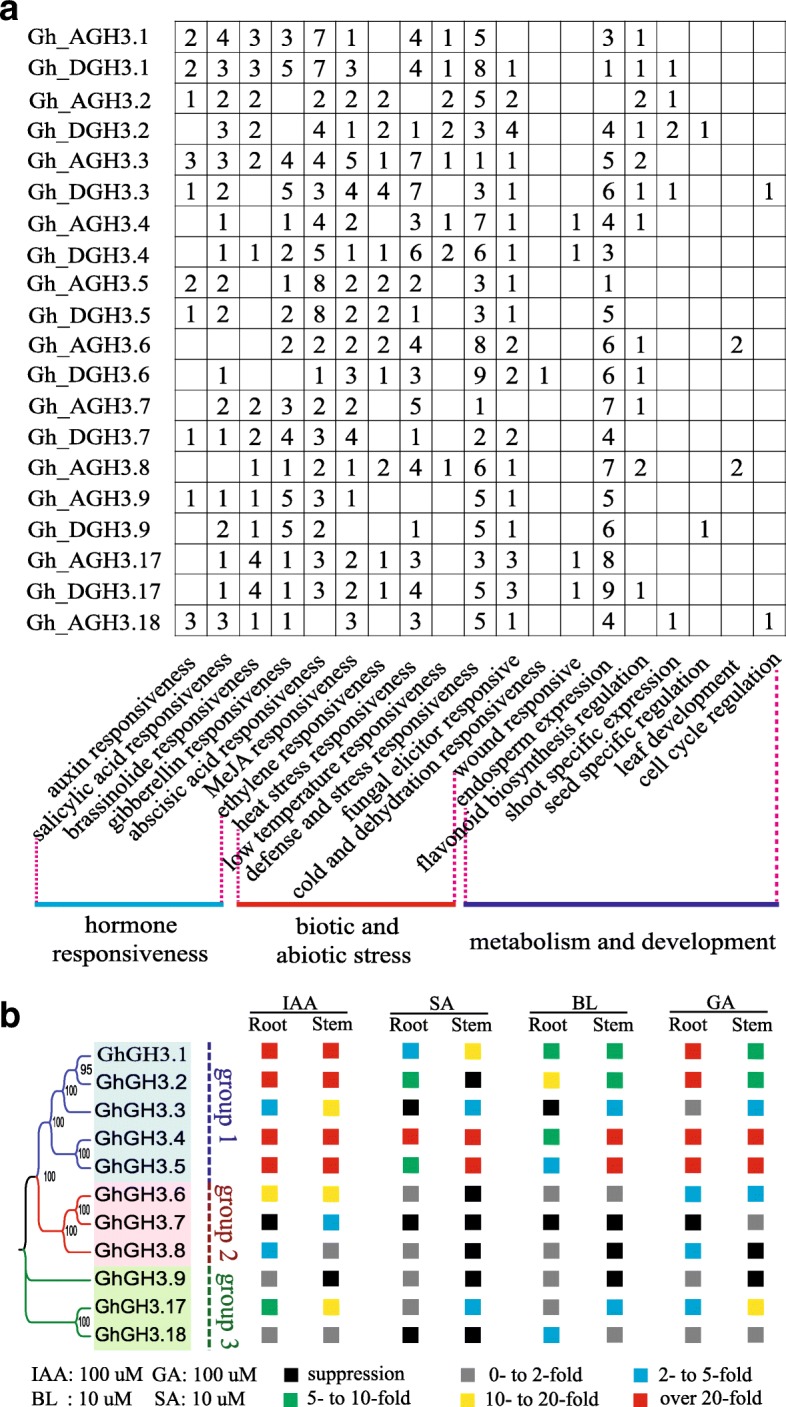
Organization of regulatory elements of *GhGH3s* and their expression in response to phytohormones. **a** The regulatory region of each *GhGH3* gene was analyzed, the numbers indicate the sum of various *cis*-acting elements response to the same stimuli. **b** The summary of *GhGH3* expression response to treatment with IAA, SA, BL and GA in roots (R) and stems (S). Each orthologous pair from At- and Dt-subgenome of *G. hirsutum* was designed as a single gene and tested together due to their highly identical sequences. In total, 11 analogue genes standing for 20 *GhGH3s* were generated and could be divided into 3 subgroups (subgroups 1–3). The expression pattern of 11 analogue genes was detected by qRT-PCR. The relative expression intensity, that is, the ratio of the highest expression level to that of the 0 h control, was allocated into six different grades ranging from suppression shown as black boxes to over 20-fold induction shown as red boxes

Gene expression is highly regulated, which is necessary for normal growth and development in plants. Precise expression patterns of candidate genes can give us important clues to their potential functions in growth and development. Trends of *GhGH3* gene expression were shown using publicly available RNA-seq data, and the 20 *GhGH3* genes displayed diverse expression patterns in 22 organs/tissues (Fig. 5a). We then verified these by quantitative RT-PCR (qRT-PCR) analysis, using custom-designed gene-specific primers (Additional file 8: Table S4). Allele pairs from the At- and Dt-subgenomes were designed as one and tested together due to the high sequence similarity of their transcription product. Thus, a total of 11 pairs of specific primers were used to check the expression profiles in roots (R), stems (S), leaves (L), flowers (FL), early developing ovules with attached fibers (1, 3, 5 OF, ovules plus fibers), and different stages of detached ovules and fibers (7, 10, 15, 20 O/F, ovules or fibers) (Fig. 5b). Based on the phylogenetic relationships and gene structure, we previously subdivided the *GH3* genes into three subgroups with the hypothesis that members from the same subgroup should have similar expression patterns as well as functions (Fig. 2a; Fig. 6b). The results of RNA-seq and qRT-PCR analysis both revealed that *GhGH3* genes were differentially expressed in various tissues/stages; unexpectedly, similar expression patterns were not observed for members of the same subgroup (Fig. 5). In our qRT-PCR experiment, *GhGH3.1* and *GhGH3.18* showed ubiquitous expression patterns in almost all tissues/stages. *GhGH3.7* exhibited high accumulation during early stages of ovule and fiber development. *GhGH3.8* is likely to be involved in the regulation of flower development and showed preferential expression in flowers. Expression levels of *GhGH3.3* and *GhGH3.6* were found to be high in vegetative organs (roots, stems and leaves). The transcripts of *GhGH3.4* and *GhGH3.5* were hardly detectable in vegetative organs but highly expressed during ovule development. Meanwhile, high transcript accumulation of *GhGH3.5* throughout the whole process of fiber cell initiation and elongation was observed. The mRNA level of *GhGH3.9* was high in a stem-specific manner, while *GhGH3.17* was predominantly expressed in flowers, 5–10 DPA (days post anthesis) ovules and 7 DPA fibers. Results were similar for most cotton *GH3* genes in RNA-seq data, and differences in expression pattern mainly existed in few organs/tissues. For example, *GhGH3.1* and *GhGH3.18* still showed ubiquitous expression patterns despite altered expression in different organs/tissues. The stem-preferential *GhGH3.3* gene showed higher expression in calycle in RNA-seq data. *GhGH3.4* had high transcript accumulation in developing fibers in addition to ovules. Although not being root-preferential and stem-specific genes, respectively, *GhGH3.6* and *GhGH3.9* also showed preferential expression in vegetative organs/tissue based on the RNA-seq data. In short, the results of analysis of RNA-seq data and qRT-PCR were basically identical, indicating the reliability and accuracy of RNA-seq data and qRT-PCR analysis, and further presenting the potential functions of *GhGH3* genes in growth and development in corresponding organs/tissues. The original RNA-seq data for *GhGH3* expression pattern analysis is supplied in Additional file 9: Table S5.

### *Cis*-acting regulatory elements in *GhGH3* promoters

Finely tuned gene expression is mainly regulated by corresponding promoters, and the action of promoters is further mediated by transcription factors through direct binding to *cis*-acting regulatory elements. Thus, analysis of upstream regulatory sequence will be helpful to understand the mode of target gene regulation and predict their potential functions [41]. To completely identify the putative *cis*-acting regulatory elements, about 3000 bp of non-coding sequence upstream of the predicted translation start site of each cotton *GH3* gene was identified, extracted and scanned. Integrated utilization of the online software tools PLACE and PlantRegMap was performed to confirm the presence of abundant regulatory cores involved in hormone, stress and development-related responses (Fig. 6a; Additional file 9: Table S6).

Many hormone-related motifs were significantly enriched in most regulatory regions of tested cotton *GH3* genes, including auxin (TGA-element, AuxRE-core), salicylic acid (TCA-element, SARE), brassinosteroid (E-box, BRRE-box), gibberellin (TATC-box, P-box and GARE-motif), abscisic acid (ABRE-box), methyl jasmonate (CGTCA-, TGACG-motif) and even ethylene (ERE-box). According to statistical analysis, two sets of stress-related motifs that are involved in abscisic acid and MeJA (methyl jasmonate) responsiveness were the most abundant *cis*-acting hormone responsive elements and were present in the promoters of all *GhGH3* genes with the exception of *Gh_AGH3.18* and *Gh_DGH3.9*, respectively. *Gh_AGH3.6* and *Gh_AGH3.8* lacked the abscisic acid responsive elements in their promoters. Both alleles of *GhGH3.2* together with *Gh_AGH3.6* showed an absence of typical gibberellin-related motifs in their regulatory regions. Interestingly, 14 out of 20 *GhGH3* genes contained BR-response elements E-box or BRRE-box. The ethylene-responsive element ERE-box was also found in the promoters of 12 *GhGH3* genes. As putative early auxin-responsive genes, intriguingly, half of the *GhGH3* promoters identified here did not show the typical auxin responsive elements TGA-element or AuxRR-core.

In addition to hormone-related motifs, a large number of stress-related elements, as expected, were observed in the promoters of cotton *GH3* genes. Specifically, defense and stress responsiveness TC-rich repeats and MBS sites can be found in all the promoters of *GhGH3* genes. In the case of temperature responsiveness, 18 *GhGH3* gene promoters contained HSE heat stress responsive elements, whereas eight possessed LTR low temperature responsive elements. Box-W1, a motif involved in fungal elicitor responsiveness, was another main component of stress-related motifs found in the promoters of all *GhGH3s* except *Gh_AGH3.1* and *Gh_AGH3.7*. Allele pairs of *GhGH3.4* and *GhGH3.17* showed the presence of a WUN-motif wound responsive element. *Gh_DGH3.6* was the only member containing the C-repeat/DRE core cold and dehydration responsive element. It is worth noting that some *cis*-elements involved in organ/tissue specific expression or metabolism, such as the Skn-1 motif, GCN4 motif for endosperm expression, as-2-box for shoot-specific expression, RY-element for seed specific regulation, MSA-like for cell cycle regulation, HD-Zip1/2 for leaf development, and MBS I/ II for flavonoid biosynthetic genes regulation, were also identified in some *GH3* promoters. All the *cis*-acting regulatory elements predicted in the upstream 3 kb regions of *GhGH3* family genes are summarized and listed in Fig. 6 and Additional file 9: Table S5, respectively. Promoter sequences of the 20 *GhGH3* genes from two individual genome assemblies (NAU-NBI, v1.1 and DOE-JGI, v3.1), which share high sequence identity, are also supplied in Additional file 10: Table S6.

### *GhGH3* gene expression in response to IAA, SA, BL and GA treatments

*GH3* genes exhibit a characteristic rapid response to active auxin within 0.25–0.5 h after treatment [11]. In addition to their typical feature of high sensitivity to auxin, these genes have also been reported to respond to SA, GA and BL (brassinolide) [10, 16]. Cotton *GH3* genes were highly conserved both in protein-coding region and organization of *cis*-acting regulatory elements. The presence of plentiful hormone-related *cis*-acting regulatory elements in *GhGH3*s promoters further increased the possibility of response to hormones as stated above. Here, we monitored the expression changes of *GhGH3* genes by qRT-PCR at five different time points (0, 0.5, 1, 3, 5 h) when exposed to 10 μM of BL and 100 μM of SA, IAA and GA. As described above, a total of 11 pairs of specific primers were employed to measure the gene expression profiles in response to hormone treatment both in roots and stems (Fig. 6b; Additional file 11: Figure S5). Under high concentration 100 μM IAA treatment, most *GhGH3* genes were induced in the early stages of treatment both in roots and stems, especially *GhGH3.1*, *GhGH3.2*, *GhGH3.4* and *GhGH3.5,* whose expressions were elevated hundreds- or even thousands-fold compared to that of the 0 h control (> 20-fold). *GhGH3.7* and *GhGH3.9* were found to be repressed in roots or stems, respectively. When exposed to SA, only *GhGH3.4* exhibited up-regulation by tens- to hundreds-fold in both roots and stems, while similar up-regulation was also observed for *GhGH3.5* in stems (> 20-fold). Transcript levels of some members, including *GhGH3.2*, *GhGH3.3*, *GhGH3.6*, *GhGH3.8*, *GhGH3.9* and *GhGH3.18*, were slightly increased or even inhibited in roots or stems (< 2-fold or inhibition). Moreover, the transcript levels of *GhGH3.7* and *GhGH3.18* were down-regulated in tested organs/tissues.

BL treatment was also found to induce expression of some *GhGH3* genes while suppressing others. The up-regulated expression of *GhGH3.4* and *GhGH3.5* was very significant in stems (> 20-fold) but moderate in roots (2- to 10-fold). Increased mRNA levels of *GhGH3.1* and *GhGH3.2* were also found at moderate levels both in roots and stems. Down-regulation of *GhGH3.7* in both tested materials was observed after BL treatment. In the case of GA treatment, the expression of *GhGH3.1*, *GhGH3.2*, *GhGH3.4* and *GhGH3.5* showed significant up-regulation both in roots and stems, whereas most other *GH3* genes exhibited only slight changes in expression level. All the results are summarized and displayed in Fig. 6 with different colored boxes. Interestingly, our qRT-PCR data showed that certain *GhGH3* genes, including *GhGH3.1*, *GhGH3.2*, *GhGH3.4*, *GhGH3.5* and *GhGH3.17*, exhibited concurrent sensitivity to all four hormone treatments with uneven expression levels.

### Expression divergence in *G. hirsutum* BR-deficient mutant *pag1*

**Fig. 7:**
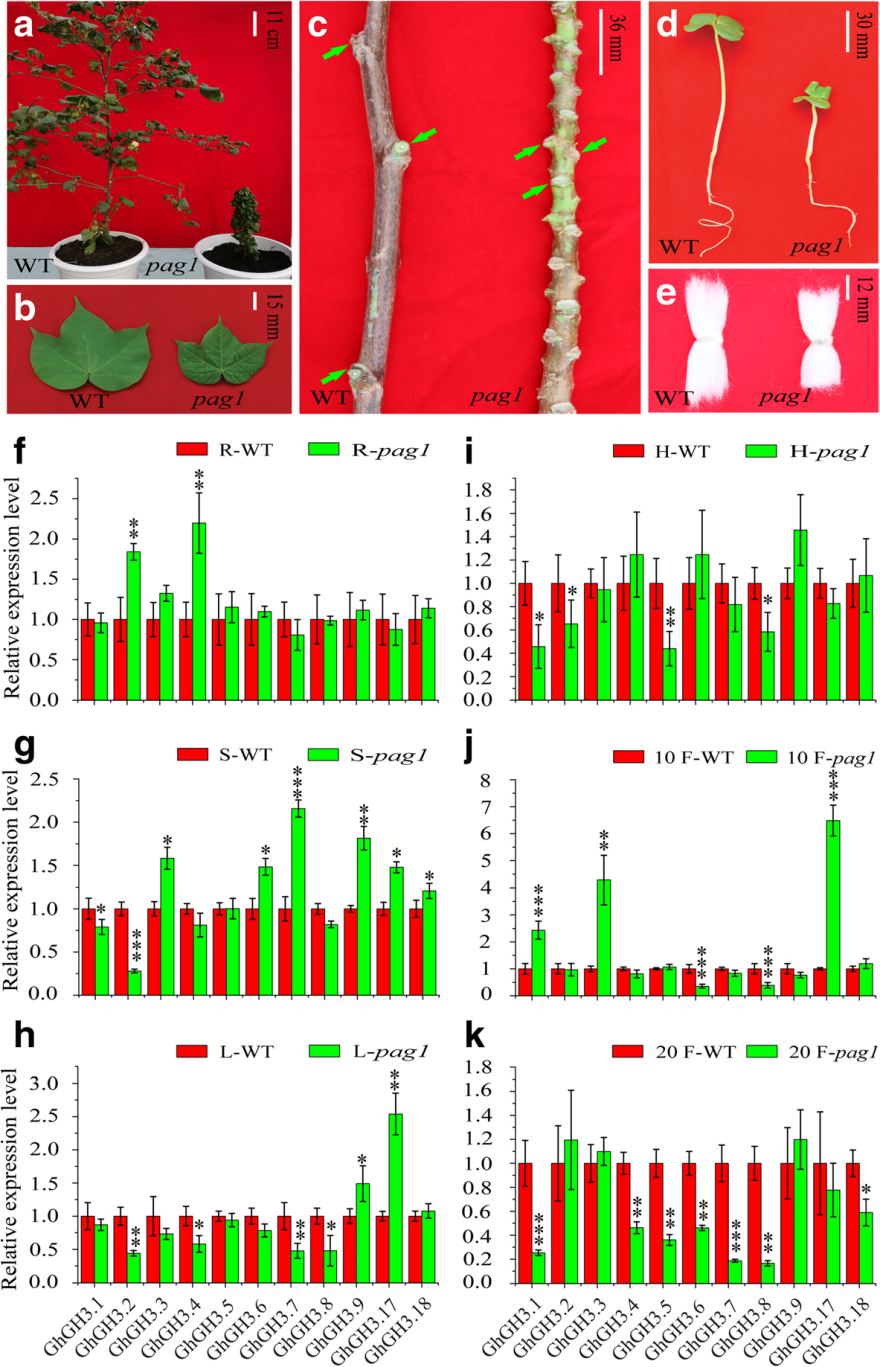
Comparison of phenotypes and expression differences in CCRI24 and *pag1*. **a** Phenotypes of WT (CCRI24) and *pag1* plants at late development stages. **b** Mature leaves. **c** Phenotypes of stems in WT and *pag1* plants. Green arrows indicate branch nodes. **d** Seedlings of WT and *pag1* plants*,* showing short hypocotyl phenotype*.* **e** Adult fiber length of WT and *pag1* plants. **f-k** the expression difference of *GhGH3* genes in root (R), stem (S), leave (L), hypocotyl (H), 10 and 20 DPA fibers in WT and *pag1* plants. The relative expression level of *GhGH3* in WT organs/tissues was designated as 1, *GhHis* 3 was used as an internal control to normalize the expression data. Error bars show standard deviation calculated from three replications. Data points marked with asterisks (* *P* ≤ 0.05, ** *P* ≤ 0.01, and *** *P* ≤ 0.001) indicate statistically significant differences between control (GS) and other tissues

*Pag1* is an artificial BR-deficient mutant derived from activation-tagged insertion in a BR metabolism-associated *P450-like* gene in the upland cotton CCRI24, which results in its over-expression and further elevated BR catabolism [42]. Compared to the wild type CCRI24, the mutant seedlings display short hypocotyls with small and wrinkled cotyledons. In the mature plant, short petioles, wrinkled leaves and shortened stems can be observed. Furthermore, the fiber length of the *pag1* mutant is reduced compared to that of WT (Fig. 7a-e). These dramatic phenotypes in turn reveal the effect of BR on cotton plant growth and development, providing a rare opportunity, along with CCRI24, to study the functions of genes downstream of BR signaling and the interaction with other hormones in cotton.

We previously analyzed BR-related *cis*-acting regulatory elements and the response of expression profiles to BL treatment. Here, we further tested expression differences of cotton *GH3* subfamily II genes between *pag1* and CCRI24. The expression pattern was monitored using qRT-PCR in roots, stems, leaves, hypocotyls, and 10 and 20 DPA fibers between the pair of materials on hand, and significant differences in expression of *GhGH3* genes were observed (Fig. 7f-k). Opposite to the elevated expression in root after BL treatment, both *GhGH3.2* and *GhGH3.4* were induced in BR-deficient mutant *pag1* compared to WT. *GhGH3.1* and *GhGH3.2* displayed down-regulated expression while *GhGH3.7* and *GhGH3.9* were up-regulated in *pag1* stem, consistent with their nature of induction or inhibition in stem when exposed to exogenous BL. Inversely, some BL-induced *GhGH3* genes in stems, including *GhGH3.3*, *GhGH3.6, GhGH3.17* and *GhGH3.18*, were unexpectedly up-regulated in *pag1* stem. The same was true for most *GH3* genes in other organs/tissues, indicating the complex regulation or expression changes in response to reduced BR in *pag1*. As described above, BR-mediated gene expression was diverse depending on the organs and developmental stages of plants. Thus, it is difficult to speculate on its expression and evaluate the results without experimental evidence of the response of *GH3* genes to BL in leaf, hypocotyl and fiber. In short, most *GH3* genes having significant difference were inhibited in the *pag1* mutant except for *GhGH3.9* and *GhGH3.17* in leaf, and *GhGH3.1, GhGH3.3* and *GhGH3.17* in 10 DPA fibers, respectively. The enhanced expression of *GhGH3.1, GhGH3.3* and *GhGH3.17* in 10 DPA fibers may be relevant to the short fiber in the *pag1* mutant by reducing IAA levels in early fiber development. Additionally, whether most *GH3* genes being inhibited in 20 DPA fibers had direct or indirect influence on fiber development in *pag1* was not clear, but it may reflect the high expression of most *GH3* genes in 20 DPA fiber of WT, which was consistent with the gradually reduced IAA level from 10 DPA. The true expression difference and the underlying molecular mechanisms need to be further investigated in the future.

## Discussion

### Conserved *GH3* subfamily II genes in cotton

The Gretchen Hagen 3 (GH3) proteins, part of the acyl-adenylate/thioester-forming enzyme superfamily, maintain hormonal homeostasis of excess IAA, SA, and JA by forming conjugates with amino acids. GH3 proteins are a small multi-gene family widely found (not restricted to plants) and have been identified in numerous plant species ranging from mosses to angiosperms [10]. In the model plant Arabidopsis, 19 GH3 proteins together with an incomplete GH3.20 are subdivided into three subfamilies, and only the members of subfamily II show IAA-amido synthetase activity [19]. Similarly, it is speculated that subfamily II members of the soybean *GH3* gene family corresponding to that of Arabidopsis have the capacity to conjugate excess auxin with amino acids based on the analysis of conserved hormone-binding residues [10]. In this study, we initially identified 17, 16, and 34 candidate genes of the *GH3* gene family in *G. arboreum*, *G. raimondii* and *G. hirsutum*, respectively (Table 1; Additional file 3: Table S2). The number of *GH3s* in *G. hirsutum* was roughly equal to the sum of that in *G. arboreum* and *G. raimondii*. This was consistent with the fact that the allotetraploid (AD1 genome) species formed from hybridization of A- and D-genome ancestors around 1–2 million years ago [31, 32]. Because the IAA-amido synthetases are restricted to members of Group II in Arabidopsis and soybean, we paid special attention to systematic bioinformatic analysis of subfamily II *GhGH3s*. Members clustering with subfamily II of the Arabidopsis *GH3* gene family were selected. Ultimately, 10, 10, and 20 candidate genes of subfamily II *GH3s* were identified in *G. arboreum*, *G. raimondii* and *G. hirsutum*, respectively. We analyzed various characteristics of *GH3* genes in cotton. Similar to *GH3s* in other plant species, most *GhGH3* genes encoded proteins with predicted molecular mass of 65–70 kDa, and isoelectric point of about 5.5–6.5 except for *GrGH3.5* [7]. Most *GhGH3* genes of subfamily II contained three exons and two introns. Members homologous to *AtGH3.9* and *AtGH3.17* seemed to have more exons, which was coherent with previous findings in maize. We also analyzed enzyme active sites according to the available crystal structures of GH3 proteins [10, 26, 43]. The residues involved in nucleotide (ATP/AMP)-binding, hormone-binding, and amino-acid preference were conserved in most *GhGH3* genes, suggesting their similar functions as IAA-amido synthetases in cotton (Fig. 3; Additional file 6: Figure S3). The numbers of *GH3* genes identified in different plants as well as in cotton are listed in Table 2. Members of subfamily III are species specific and have only been identified in Arabidopsis to date. Interestingly, the proportion of subfamily II *GH3* genes to all *GH3* members is relatively stable ranging from 0.4 to 0.67 in observed plants, indicating strict control of *GH3* gene number in different genomes.

**Table 2:** Proportion of subfamily II *GH3s* in various plant species

species	I	II	III	total	ratio	Ref.
*G. arboreum*	7	10	–	17	0.59	–
*G. raimondii*	6	10	–	16	0.63	–
*G. hirsutum*	14	20	–	34	0.59	–
*A. thaliana*	2	8	10	20	0.40	[20]
*S. lycopersicum*	9	6	–	15	0.40	[15]
*M. truncatula*	10	7	–	17	0.41	[16]
*G. max*	12	16	–	28	0.57	[10]
*O. sativa*	5	7	–	12	0.58	[4, 12]
*Z. mays*	5	8	–	13	0.62	[4]
*V. vinifera*	3	6	–	9	0.67	[13]
*M. domestica*	5	10	–	15	0.67	[17]
*S. bicolor*	7	9		16	0.56	[3]

### Diverse expression pattern of *GH3* genes in cotton

Determining spatial and temporal mRNA expression patterns of candidate genes can partly predict their biological functions in tissue/organ development. Numerous studies of *GH3* gene expression patterns in various plants provided us with abundant reference data for analyzing *GH3* expression in cotton [8, 10, 12–16]. In this study, the transcript level of *GhGH3s* was measured in 15 tissues/stages of vegetative organs, developing ovules and fibers. Similar to other plant species, cotton *GH3* genes were differentially transcribed in a tissue-, stage-, and cell-specific manner. Some of the *GhGH3* genes, including *GhGH3.4, GhGH3.5, GhGH3.7, GhGH3.8, GhGH3.9* and *GhGH3.17*, showed low expression levels in leaves (Fig. 4b). This observation is similar to previous findings that all the *ZmGH3s* (*Z. mays*) and most of the *MtGH3s* (*M. truncatula*) display lower expression levels in leaves as compared with other organs [16]. Many *SlGH3* (*S. lycopersicum*) genes also show lower expression levels in leaves [15]. Besides this, the accumulation of these *GhGH3* mRNAs was low either in roots or stems, indicating their limited role in vegetative organ development. In Arabidopsis, *ydk1-D* and *dfl1-D*, two T-DNA insertion activation tagged mutants, show shortened primary roots and reduced lateral root number due to over-expression of *AtGH3.1* and *AtGH3.6*, respectively [28, 29]. *GhGH3.1* and *GhGH3.6,* the orthologs of *AtGH3.1* and *AtGH3.6*, respectively, in cotton exhibited higher expression in roots, suggesting their possible role in root development.

In rice, eight out of 12 *GH3* genes are predominately expressed in flowers [8]. *GhGH3.8* displayed its highest expression level in flower, suggesting a role in flower development. In the process of fiber initiation and development, auxin undoubtedly is an important phytohormone that regulates fiber initiation, number and length [35]. Some *GhGH3* genes like *GhGH3.4*, *GhGH3.5 and GhGH3.7*, exhibited high accumulation in ovules with attached fibers or in fibers. The enrichment of *GH3* transcripts in certain cells may mediate free IAA content, thereby further controlling ovule and fiber development or acting as the signaling stimuli to regulate the transition from one stage to another. Cotton has a perennial and indeterminate growth habit and excessive vegetative growth can have a negative impact on cotton production. Cotton canopy is usually controlled by applications of excess plant growth regulator mepiquat chloride (1,1-dimethylpiperidinium chloride) [44]. In Arabidopsis, over-expression of most *GH3* genes in subfamily II alters the homeostasis of active auxin, resulting in a dwarf phenotype. This gives us an important clue that cotton canopy could be controlled by modulating *GH3* expression. Our qRT-PCR data show that *GhGH3.1, GhGH3.3* and *GhGH3.18* are predominantly expressed, and *GhGH3.9* is specifically expressed in stem, indicating their possible roles in cotton growth and development.

### Regulatory element organization implies *GH3* family relevance to various stimuli

Gene expression or transcription is initiated through the action of an upstream regulatory promoter region, which can be considered as the combination of many *cis*-acting regulatory elements fused with a minimal basic start unit. Various combinations of regulatory cores confer the promoters with characteristics of strength, time-space specificity and response to stimuli. Therefore, analysis of regulatory elements of a target gene promoter can help us to predict its expression in response to various stimuli. Scanning of *GhGH3* gene family promoters revealed the presence of numerous phytohormone-, stress- and development-related regulatory elements. AuxRE and TGA-element are two auxin-response regulatory elements. Previous studies have shown that the presence and absence of AuxRE can be correlated with inducibility or lack thereof for most *GH3* genes [15, 27, 45]. The majority of *GH3* gene promoters contain one or multiple AuxREs, suggesting their possible response to auxin [15, 16]. In cotton, five out of 20 *GhGH3* genes contain AuxREs and half of them contain at least one AuxRE or TGA-element, implying their possible response to auxin. In addition to auxin responsive elements, the regulatory cores required for other growth-promoting hormone (BL and GA) responses were also widely distributed in the promoters of the *GhGH3* gene family. E-box and BRRE are typical *cis*-elements that can be recognized by transcription factors downstream of the BR signaling pathway, BES1 or BZR1 [46]. In Arabidopsis, independent chromatin-immunoprecipitation microarray (ChIP-chip) experiments found that some members of *GH3* gene family are the direct targets of BES1 or BZR1 [47, 48]. In sorghum and tomato, the expression of some *GH3* genes in response to BRs has been confirmed [15]. These results suggest that BRs might directly or indirectly regulate the expression of some *GhGH3* genes, as they harbor the *cis*-elements required for BR response. In the case of GA, the molecular mechanism by which some *GH3* genes are regulated by the GA signaling pathway is not clear, but the real response of some *GH3* genes to GA in tomato cannot be ignored [15]. We therefore present the possibility that GA response elements (TATC-, P-box and GARE-motif) enriched in the promoters of some *GhGH3* genes may confer their response to GA. The ethylene response element ERE also can be seen in most *GhGH3* gene family promoters, consistent with findings in pepper and tomato [15, 49]. The plentiful predicted hormone-related *cis*-acting regulatory elements that exist in most *GhGH3* promoters indicate their important roles as core regulators in many hormone signaling pathways.

In addition to maintaining auxin homeostasis, *GH3* gene family members are widely involved in disease resistance, biotic and abiotic stress responses, consistent with the enrichment of numerous stress-related *cis*-elements [3, 9, 24]. *GhGH3* promoters contain many stress-related regulatory cores, including SA-responsive TCA-elements and SARE, ABA-responsive elements ABRE, MeJA-responsive elements CGTCA- and TGACG-motif, abnormal temperature-related elements HSE and LTR, defense and stress responsive elements TC-rich repeats and MBS, wound-responsive elements WUN-motif, and fungal elicitor responsive elements Box-W1. Among them, SARE and TCA-element, two typical SA-responsive *cis*-acting elements, were observed in 18 promoters out of 20 *GhGH3* genes. Salicylic acid is a primary growth hormone that mediates plant disease resistance and abiotic stress responses [24, 50, 51]. Most established *GH3* genes are more or less responsive to SA [4, 14–17]. Notably, some organ/tissue specific elements involved in shoot, seed, leaf or endosperm development were also observed. Except for *Gh_AGH3.2*, all members contained the endosperm expression elements Skn-1- and GCN4-motif. The promoters of *Gh_DGH3.2* and *Gh_DGH3.9* had the seed-specific regulation core RY-element, implying their potential functions in seed development. Some *GhGH3* genes containing the shoot-specific expression element as-2-box are worth further study for their possible roles in shoot development.

### Variable response of GH3 family to IAA, SA, BL and GA

Analysis of *cis*-acting regulatory elements showed large numbers of regulatory cores in the promoters of the *GhGH3* gene family. To investigate their particular response to different hormones, qRT-PCR analysis was conducted to monitor the expression changes of *GhGH3* genes in root and stem in response to IAA, SA, BL and GA. IAA treatment sharply up-regulated most *GhGH3* genes, slightly inducing some members while repressing others in roots or stems. Similar expression trends have been observed in previous reports of the *GH3* gene family in maize and sorghum [3]. In maize, the expression pattern of *ZmGH3s* responsive to auxin in root is different to that in stems. These differences include the induction intensity and the expression pattern of up-regulation or suppression. GH3s, especially the members of group II, are mainly involved in auxin homeostasis and stress response. The expression differences reveal subtle regulation of transcriptional levels of *GhGH3* genes to maintain the free IAA content at an appropriate level for normal growth and development or in response to stress. Similar to IAA treatment, intricate expression differences can be seen on exposure to SA, BL and GA. In contrast, most *GH3* genes are up-regulated in maize, *M. truncatula* (clover) and apple, whereas the majority of *GH3* genes are down-regulated in tomato on exposure to SA treatment [3, 15–17]. Most of the tested *GhGH3* genes were up-regulated in response to GA, however, these results are opposite to those in tomato [15]. The altered expression response to certain hormones may be due to the difference in plant species, suggesting novel functions in adaptation to changed circumstances in the process of evolution. In brief, all four tested hormones seemed to regulate the expression of *GH3* genes in both a positive and negative manner in cotton. However, the response to hormones in roots and stems was different. Notably, we previously subdivided the genes of subfamily II into three subgroups based on their evolutionary relationships (Fig. 2a; Fig. 6b). Our qRT-PCR data show that the majority of subgroup 1 genes appeared to have higher sensitivity to stimuli. When treated with IAA, SA, GA or BL, the expression of most members of subgroup 1 was significantly up-regulated as compared to members of subgroup 2 and 3 (over 20-fold). These findings suggest strong functions for subgroup 1 members in growth and development, as well as in response to environmental stresses.

### GH3s mediate fiber development, plant architecture, seed or somatic embryogenesis

GH3s, a small multi-gene family designated as the early auxin-responsive gene family, are widely distributed in higher plants. The particular function of subfamily II *GH3* genes is to maintain auxin homeostasis by converting excess auxin to amino acid conjugates leading to inactivation or degradation [19]. Due to significant functional redundancy, loss-of-function mutations in one gene of subfamily II usually do not exhibit any visible phenotype or altered sensitivity to IAA. However, over-expression of one functional gene of subfamily II usually results in small or dwarf phenotypes across whole activation-tagged mutant or overexpression lines [25, 27–30, 36]. Previous findings imply that the dramatic effect on plant phenotype is caused by increased IAA-amido synthetase activity, which results in the reduction of free auxin content [30, 36, 52]. Notably, although the expression level of *AtGH3.6* was enhanced by the activation-tagged insertion, free IAA content of *dfl1*-*D* was not significantly decreased [19]. In maize, most *ZmGH3* genes were up-regulated in shoots in response to MeJA with no effect on free IAA content. These opposite findings may be attributed to small undetected or limited decline of free IAA in important tissues/organs. In addition, some other molecular mechanisms underlying this phenomenon may also exist and need further study. Taken together, *GH3* gene expression changes usually alter free IAA content, with effects on plant growth and development as well as architecture.

Practical applications in the textile industry and as a model for studying single cell development make cotton a distinct crop plant. Numerous efforts have been made to discover the molecular mechanisms of fiber cell initiation and development in order to improve cotton fiber quality. Fiber cells are derived from ovule epidermal cells and their initiation and development seem to be more related to ovules themselves [53]. Ovules and fiber cells initiate accumulation of auxin at − 1 and 0 DPA, respectively, peak at + 2 DPA and gradually decrease to basal levels by + 10 DPA [35, 54]. This is consistent with the increasing expression of *GhGH3.4* and *GhGH3.5* with peaks also at 10 DPA, which may degrade and/or store excess IAA. In addition, enhanced expression of *GH3* genes was not reported to have significant influence on seed morphology in Arabidopsis. Analogously, although dwarf plants and short siliques can be seen, transgenic Arabidopsis plants overexpressing *GhGH3.5* gene do not display obvious changes in seed morphology (data not shown), and this may be the same case for cotton. The fact that *GhGH3.4*, *GhGH3.5* and *GhGH3.7* show high expression levels in developing ovules raises an interesting question about their biological function in ovule or fiber development. As accumulated IAA in fiber initials depends mainly on efflux transport from ovules rather than in situ synthesis, we speculate that some *GhGH3* genes may play a role in fiber initiation and development by regulating the IAA content of ovules. Further, stable genetic transformation is necessary for functional characterization of these genes to explore their specific roles. Somatic embryogenesis is, to date, the most popular and stable transformation approach for cotton. However, this process is time-consuming, expensive, and labor-intensive, so that progress in increasing the regeneration rate has been limited [55]. The morphological and physiological characteristics of zygotic embryos are similar to those of somatic embryos, sharing sets of genes with similar expression patterns [56, 57]. Up-regulation of some *GhGH3* genes during ovule development may imply their functions in seed or somatic embryo development, either directly or indirectly via altered free IAA content. Previous studies showed that over-expression of *GH3* genes can alter plant architecture directly by local and/or systemic reduction of biologically active auxin in Arabidopsis and rice [24, 28–30, 36]. This is consistent with the most recent finding that low-level auxin zones in shoot apical meristem (SAM) organization center could break stem cell homeostasis and promote their proliferation, resulting in enlarged SAM and more lateral organs [58]. In this study, *GhGH3.3* and *GhGH3.9* were found to be expressed in a stem-preferential or -specific manner, thus we speculate that these *GhGH3* genes together with others could be potential regulators for genetic modification of plant architecture in cotton. Finally, the presence of BR- and GA-related *cis*-acting regulatory elements and their particular response suggest that some *GH3* genes might be involved in BR and GA signaling pathways [15]. We extrapolate that some dramatic phenotypes in BR- or GA-deficient mutants might be due to the altered expression of *GH3* genes, which in turn reveal the applicability of *GH3* genes to genetic modification of cotton plant architecture. In addition to the potential functions in architecture and development, as described above, cotton *GH3* genes of subfamily II as well as I are also expected to be candidate genes for adversity-related studies and applications in cotton genetics and breeding.

## Conclusion

In this study, a total of 10, 10, and 20 *GH3* subfamily II genes were identified in *G. arboreum*, *G. raimondii*, and *G. hirsutum*, respectively. *GhGH3* genes identified here clustered into subfamily II of the *GH3* gene family and were conserved in intron numbers, protein motifs, active sites, and proportion in cotton *GH3* genes. Expression pattern analysis showed that cotton *GH3* genes were differentially expressed, suggesting their diverse roles in cotton plant growth and development. Promoter *cis*-element analysis and expression patterns under hormone treatments illustrated that cotton *GH3* genes were also responsive to various stimuli. The variable expression of *GhGH3* genes in BR-deficient mutant *pag1* further confirms the conclusion stated above. Based on our experimental results, we conclude that some members of *GhGH3* genes could be possible mediators for the study of cotton fiber development, fiber improvement, seed development, reconstruction of plant architecture, somatic embryogenesis, and stress tolerance. In short, our work provides comprehensive information on subfamily II of the cotton *GH3* gene family to aid in future exploration of their functional divergence and genetic manipulation.

## Materials and methods

### Plant materials, growth conditions, and hormone treatments

*G. hirsutum* cultivar CCRI24, grown under experimental field conditions, was used to perform the expression of candidate genes. Flower buds were tagged or collected on the flowering day recorded as 0 DPA. The 7, 10, 15, and 20 DPA ovules and fibers were collected from the tagged buds. 1, 3, and 5 DPA fibers were sampled together with ovules because of the difficulty of separating them from each other. Young roots, stems and leaves were also collected from plants grown in the field after washing three times using distilled water. In order to compare *GhGH3* expression differences between CCRI24 and *pag1*, 10 and 20 DPA fibers were collected as described above from the BR-deficient mutant *pag1* that was grown simultaneously in the experimental field under normal conditions. The remaining materials were collected from sand-grown seedlings. In detail, hypocotyls of CCRI24 and *pag1* were obtained from seedlings at the flattened-cotyledon stage. Materials of roots, stems and leaves were collected from CCRI24 and *pag1* at the three-leaf stage. All samples were wrapped with DNase/RNase-free silver paper or tubes and frozen in liquid nitrogen immediately and stored at − 80 °C for subsequent use.

For phytohormone treatment, the CCRI24 seeds were planted in sand containers kept in a growth chamber at 28 °C with a photoperiod of 16 h light and 8 h dark. Flattened cotyledons were uprooted from the sealed container and washed with sterile water, then transferred into Hoagland’s solution with continuous air supply through an oxygen pump. Opaque containers were used and the nutrient solution was changed every 3 days until the third true-leaf stage. Seedlings were then immersed in new hydroponic culture solutions supplied with 100 μM IAA, SA, GA, or 10 μM BL. Root and stem samples at 0.5-h, 1-h, 3-h, and 5-h after applications of hormones were collected for qRT-PCR experiment. All samples were quick-frozen in liquid nitrogen and stored at − 80 °C for RNA isolation.

### RNA extraction, cDNA synthesis, and qRT-PCR analysis

Total RNA was isolated using RNAprep Pure Plant kit (TIANGEN, Beijing, China). To avoid degradation all steps were carried out at low temperature. RNA quantity and quality was determined using both Nanodrop2000-C spectrophotometer (Thermo Fisher Scientific, Wilmington, DE, USA) and gel electrophoresis. Approximately 1 μg of qualified total RNA of each sample was used to synthesize first strand complementary DNA according to the manufacturer’s instructions of the PrimeScript® RT Reagent kit after gDNA Eraser treatment (TaKaRa, Dalian, China).

Gene-specific primers for qRT-PCR were designed using the NCBI Primer-Blast Tool. Detailed information of all the primers used in this study is listed in Additional file 8: Table S4. qRT-PCR analysis was performed on a LightCycler TM 480 II System (Roche Applied Science, Basel, Switzerland) using LightCycler 480 SYBR green 1 Master mix (Roche). The relative amounts of candidate genes were calculated with the 2^-ΔΔCT^ method. The cotton histone3 (GenBank: AF024716.1/locus_ID of NAU-NBI, v1.1: Gh_D03G0370) gene was used as the reference gene. All qRT-PCRs were performed in triplicate.

### Genome-wide identification and nomenclature of GH3 subfamily genes in three cotton species

Available reference genome sequences, including *G. hirsutum* (NAU-NBI, v1.1; DOE-JGI, v3.1), *G. arboreum* (BGI, v2.0), and *G. raimondii* (BGI-CGP, v1.0; JGI, v2.0) were downloaded from COTTONGEN (http://www.cottongen.org/) or JGI (https://phytozome.jgi.doe.gov/pz/portal.html). Twenty protein sequences of the *GH3* gene family of Arabidopsis were taken from the Arabidopsis Information Resource (TAIR, http://www.Arabidopsis.org/). Firstly, the Hidden Markov Model profile (PF03321.10, GH3 auxin-responsive promoter) of Arabidopsis *GH3.1* (AtGH3.1) downloaded from pfam database (http://pfam.xfam.org/) was utilized as the query to search against the proteomes of the three cotton species using local HMM search program HMMER3.0. All genes with E-values below the threshold value of 10^− 5^ obtained from these three searches were presumed to be tentative genes for amino acid sequence retrieval by local blastdbcmd program (blast-2.2.31+). Further, the online Simple Modular Architecture Research Tool (SMART; http://smart.embl-heidelberg.de/) was employed to test the presence of a GH3 domain in all the candidate genes. Finally, in order to identify the orthologous genes of subfamily II of Arabidopsis GH3s in the three cotton species, each set of tentative cotton GH3s was aligned with the 20 GH3s of Arabidopsis using ClustalX 1.83 and three neighbor-joining (NJ) phylogenetic trees were constructed independently using MEGA7.0 (http://www.megasoftware.net/) or PHYLIP 3.6 (http://evolution.genetics.washington.edu/phylip.html). Bootstrap values were calculated from 1000 iterations. Only the genes that clustered with group II of the Arabidopsis GH3 gene family were considered theoretically as the group II genes of cotton and selected for further analysis.

### Systematic bioinformatic analysis of cotton GH3 II subfamily

For phylogenetic analysis of cotton GH3 subfamily II with other plant species, proteomes of *G. max* (v9.0), *V. vinifera* (Genoscope.12X), *S. lycopersicum* (iTAGv2.40), *Z. mays* (v10), and *O. sativa* (v10) were downloaded from JGI (https://phytozome.jgi.doe.gov/pz/portal.html). All the GH3 protein sequences were obtained directly from the supplemental materials or retrieved from the proteomes based on the gene locus provided by previous studies. Similarly, each set of GH3s of the five plant species was aligned with the 20 GH3s of Arabidopsis; only the orthologous genes of subgroup II of the Arabidopsis GH3 family were selected. A neighbor-joining phylogenetic tree was constructed as mentioned above and visualized using Evolview (http://www.evolgenius.info/). For genetic structure analysis, the GFF (General Feature Format) files of 20 *GhGH3* genes were retrieved from the genome database of *G. hirsutum* (NAU-NBI, v1.1). Intron-exon organization was then visualized using the online Gene Structure Display Server 2.0 (GSDS 2.0) (http://gsds.cbi.pku.edu.cn/). Conserved motifs were predicted by the online program MEME (http://meme-suite.org/). RNA-seq data were downloaded from the NCBI Sequence Read Archive (https://www.ncbi.nlm.nih.gov/bioproject/PRJNA248163/) and analyzed as previously reported [59]. A heatmap was drawn using online software omicshare (http://www.omicshare.com/tools/Home/Soft/heatmap). The length, molecular weight, and isoelectric point of each GH3 protein was calculated using ExPasy (https://web.expasy.org/protparam/). Multiple sequence alignment of the enzyme active sites of selected GH3 protein sequences in Arabidopsis (8 AtGH3s), soybean (8 GmGH3s), and cotton (20 GhGH3s) was carried out using the local alignment tool of MAFFT7 (http://mafft.cbrc.jp/alignment/software/). The resulting output was then imported and visualized using the online tool ESPript (http://espript.ibcp.fr/ESPript/cgi-bin/ESPript.cgi). Chromosomal distribution analysis was conducted using Mapchart (http://mapchart.software.informer.com/). CIRCOS software was employed to draw a collinearity map [59]. Analysis of *cis*-acting regulatory elements was performed using plantCARE (http://bioinformatics.psb.ugent.be/webtools/plantcare/html/) and PlantRegMap (http://plantregmap.cbi.pku.edu.cn/binding_site_prediction.php).

## Additional files

Additional file 1: **Table S1.** All protein sequences used in this study. (XLSX 71 kb)

Additional file 2: **Figure S1.** Three NJ phylogenetic trees for identification of *GH3* genes in cotton. *GH3s* in *G. arboreum* (a)*, G. raimondii* (b) and *G. hirsutum* (c) are shown*.* All *GH3s in* Arabidopsis are marked by solid red circles, members of subfamily II *GH3s* in Arabidopsis are highlighted in lime. Gene name is located on the right side of the locus_ID. (PDF 1002 kb)

Additional file 3: **Table S2.** Characteristics of subfamily II *GH3s* in *G. arboreum* and *G.raimondii.* Detailed characteristics of *GH3s* in two diploid cottons are shown. Locus_ID and sequence of *GrGH3.5* in *G. raimondii* (BGI, v1.0) was replaced by that of its analogue in *G. raimondii* (JGI, v2.0) due to the abnormal length possibly caused by improper genomic assembly. (DOCX 24 kb)

Additional file 4: **Table S3.** Comparison of characteristics of *GH3s* identified in two genome assemblies of *G. hirsutum.* (DOCX 19 kb)

Additional file 5: **Figure S2.** Sequence logos of 18 motifs. (EPS 2278 kb)

Additional file 6: **Figure S3.** Multiple sequence alignment of 20 *GhGH3s*. (PDF 35 kb)

Additional file 7: **Figure S4.** Chromosomal distribution of subfamily II *GhGH3* genes together with putative subfamily I *GhGH3* genes. The orthologous pairs are linked with red dotted lines. Gray locus_IDs, the putative *GhGH3* genes of subfamily I in *G. hirsutum*, are also mapped to the chromosomes. (EPS 1142 kb)

Additional file 8: **Table S4.** Primers used in this study. (DOCX 23 kb)

Additional file 9: **Table S5.** The original RNA-seq data for *GhGH3* expression pattern analysis. (XLSX 20 kb)

Additional file 10: **Table S6.** Organization of *cis*-acting regulatory elements in the promoters of subfamily II members of cotton *GH3* gene family. The number represents the upstream distance to start codon. “+” and “-” represent the plus strand and minus strand, respectively. (XLSX 84 kb)

Additional file 11: **Figure S5**. Expression of *GhGH3* gene family in response to IAA, SA, BL and GA treatment. R, roots; S, stems. The expression pattern of each *GhGH3* gene in response to IAA, SA, BL and GA treatment is also shown. (PDF 1630 kb)

## Abbreviations

*A. thaliana*: *Arabidopsis thaliana*
DPA: Days Post-Anthesis
*G. arboreum*: *Gossypium arboreum*
*G. hirsutum*: *Gossypium hirsutum*
*G. max*: *Glycine max*
*G. raimondii*: *Gossypium raimondii*
GH3: Gretchen Hagen 3
*M. truncatula*: *Medicago truncatula*
*O. sativa*: *Oryza sativa*
*S. lycopersicum*: *Solanum lycopersicum*
*V. vinifera*: *Vitis vinifera*
*Z. mays*: *Zea mays*
