# Supplementary figures and images for: Genome-wide analysis of cotton GH3 subfamily II reveals functional divergence in fiber development, hormone response and plant architecture
Source: BMC Plant Biol. 2018 Dec 12;18:350. doi: 10.1186/s12870-018-1545-5 (PMC6291927; doi:10.1186/s12870-018-1545-5)

a

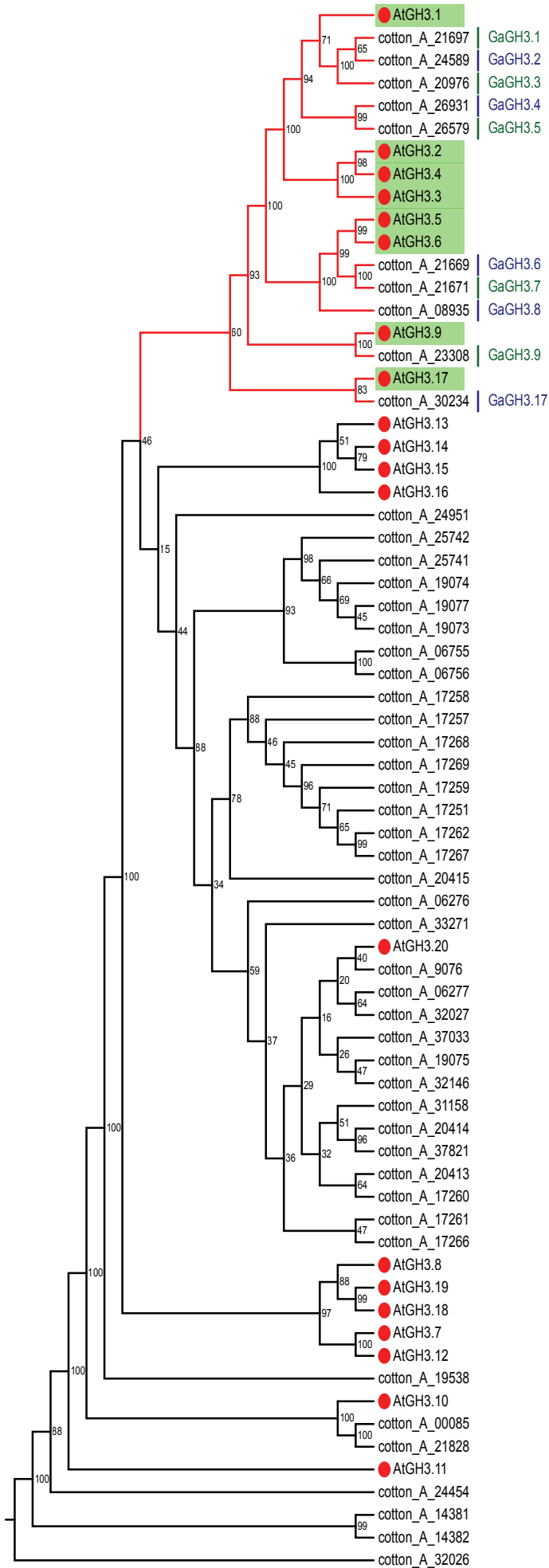

b

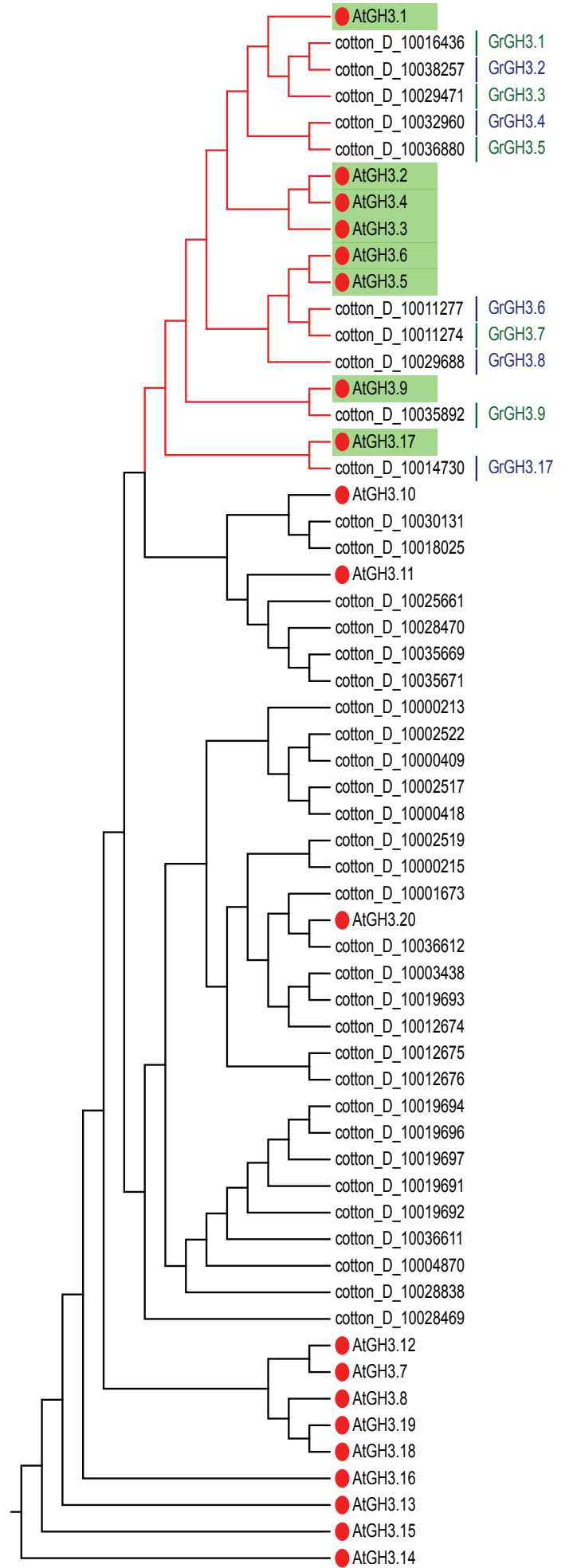

Supplement: Supplementary file 2 — Figure S1. Three NJ phylogenetic trees for identification of GH3 genes in cotton. GH3s in G. arboreum (a), G. raimondii (b) and G. hirsutum (c) are shown. All GH3s in Arabidopsis are marked by solid red circles, members of subfamily II GH3s in Arabidopsis are highlighted in lime. Gene name is located on the right side of the locus_ID. (PDF 1002 kb) [file 12870_2018_1545_MOESM2_ESM.pdf]

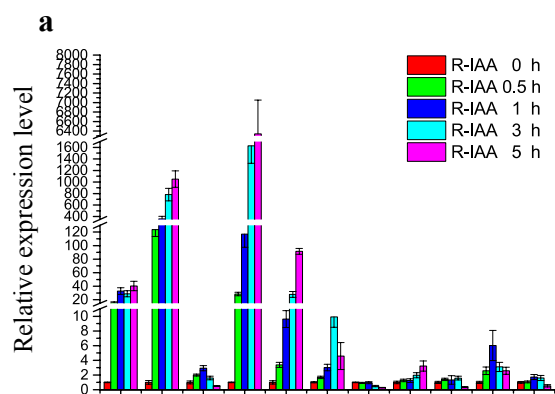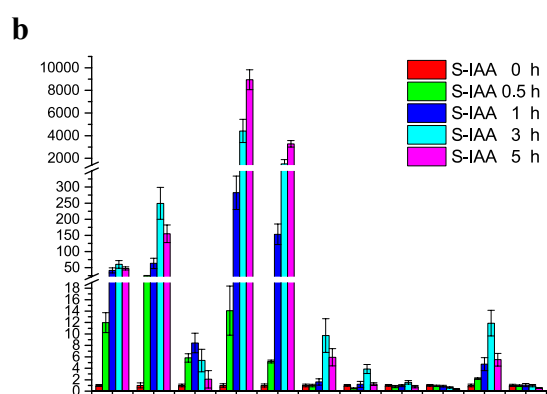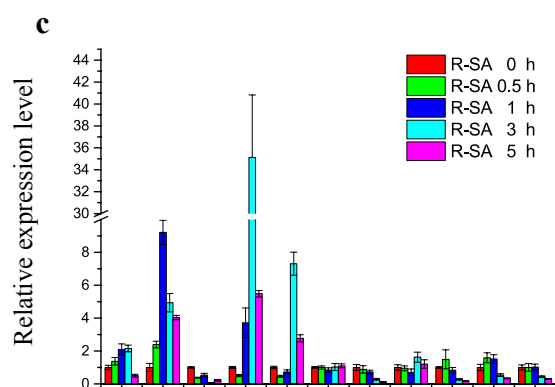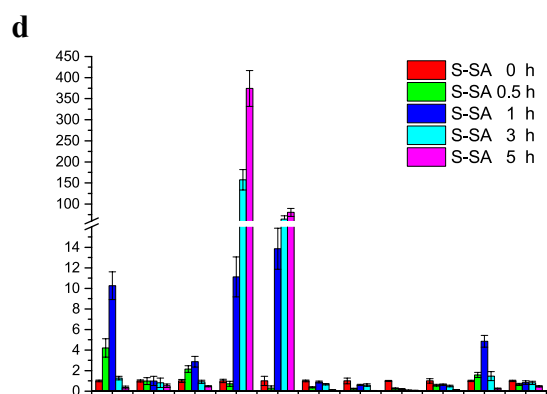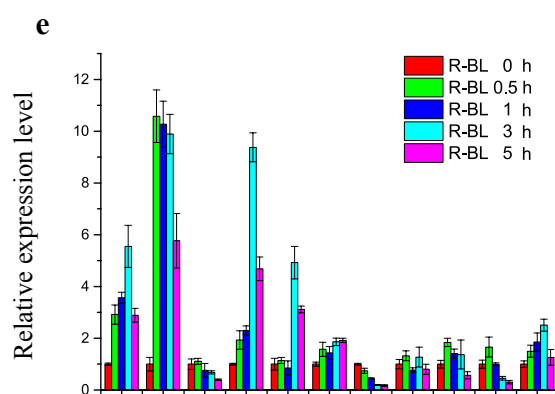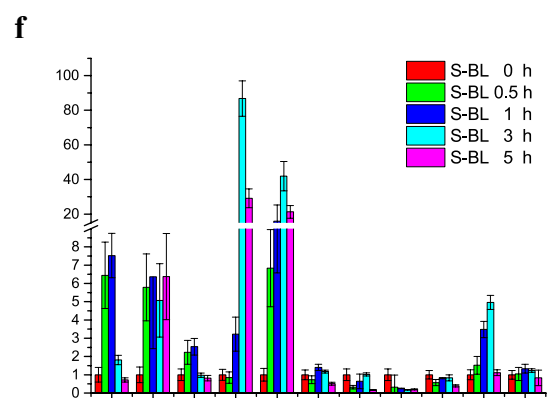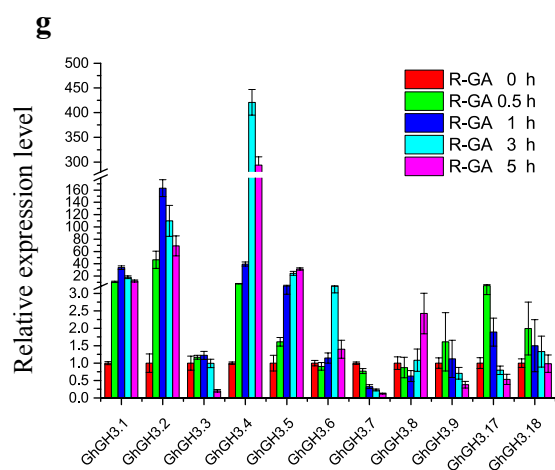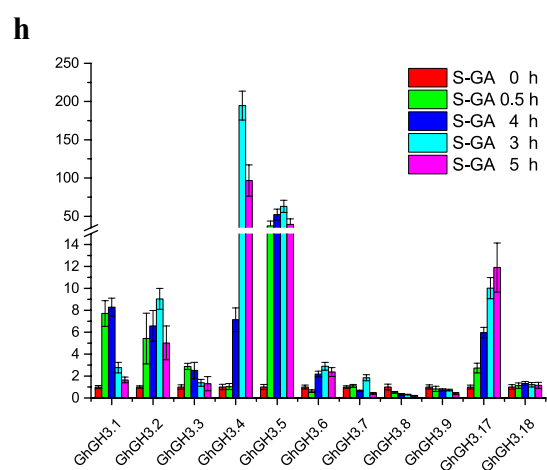

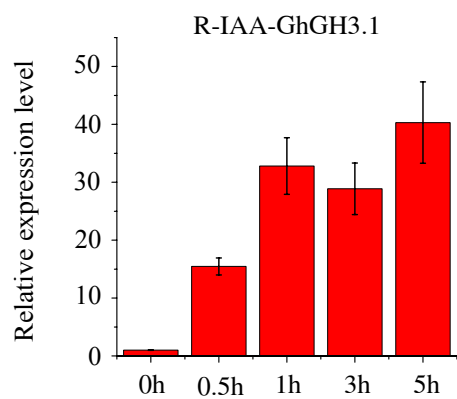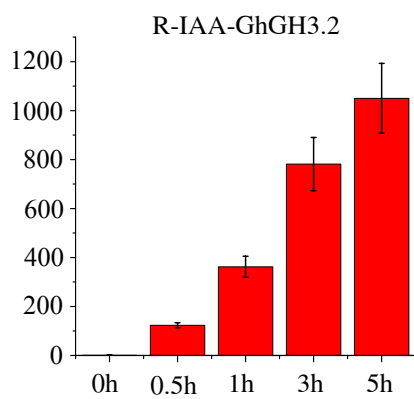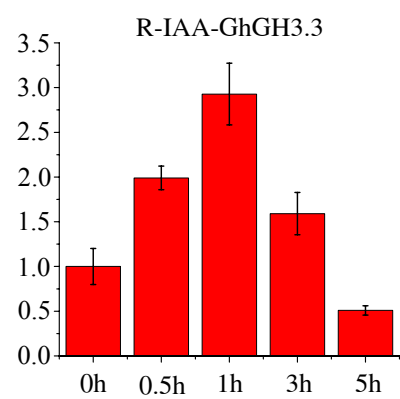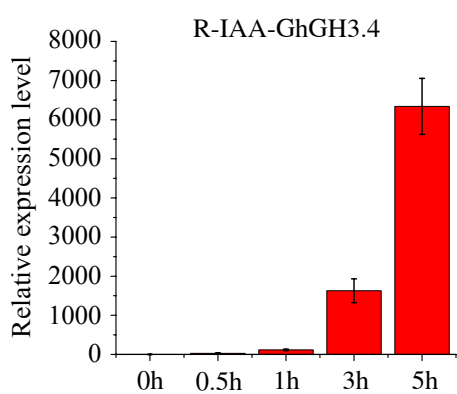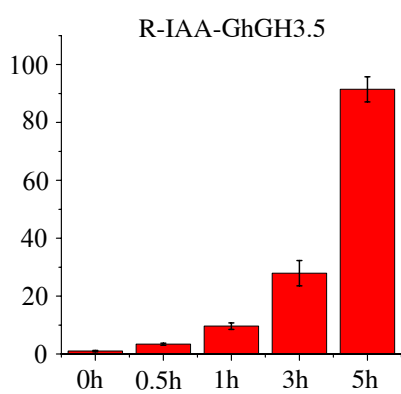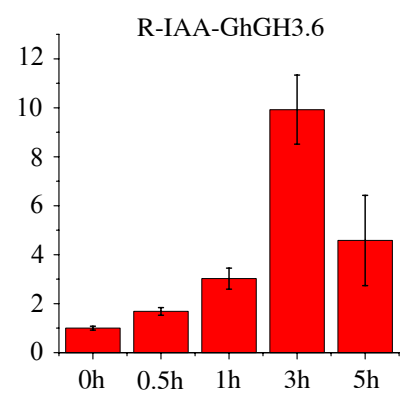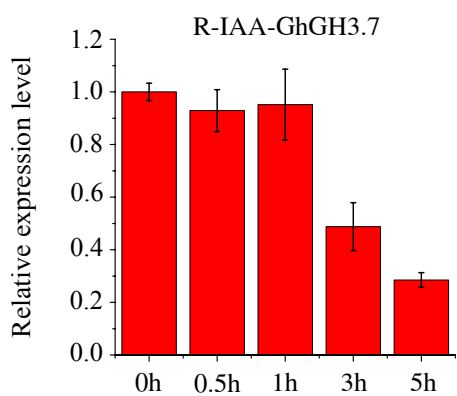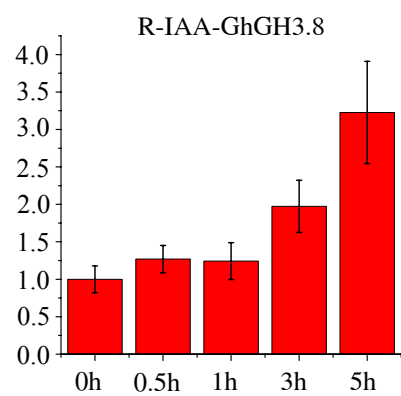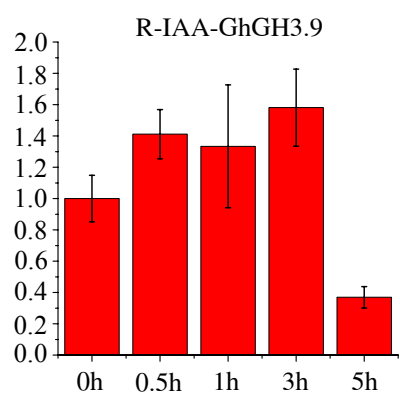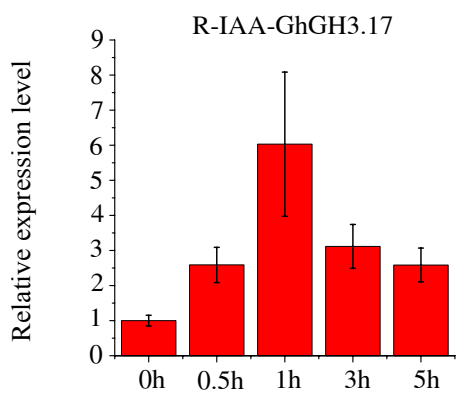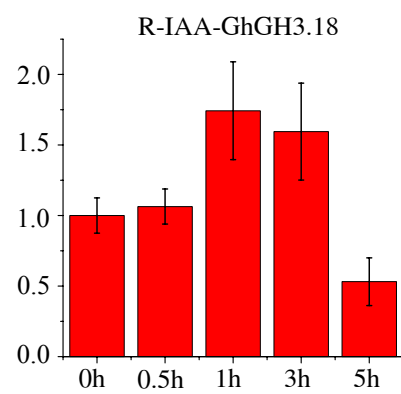

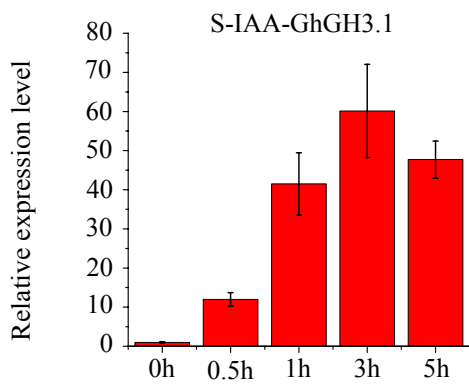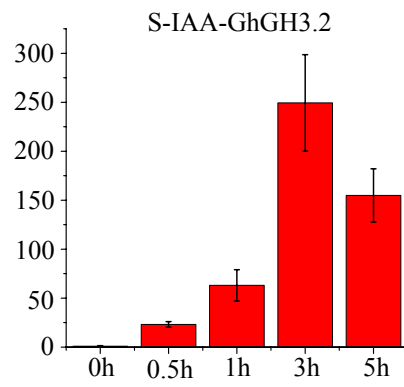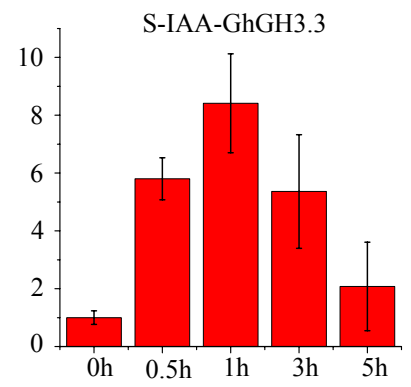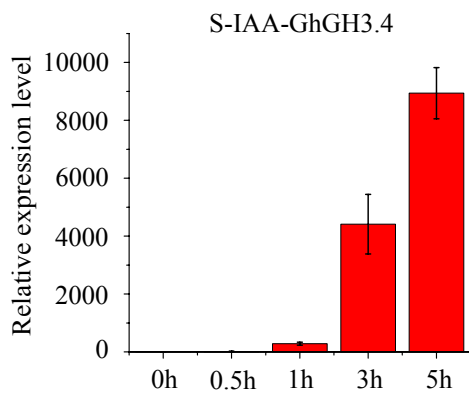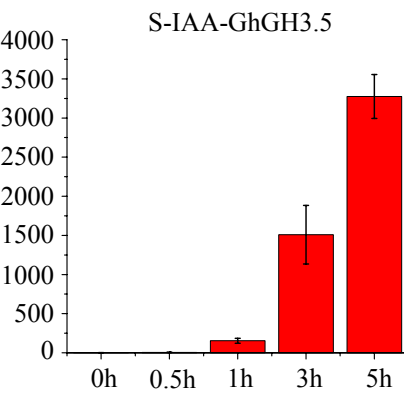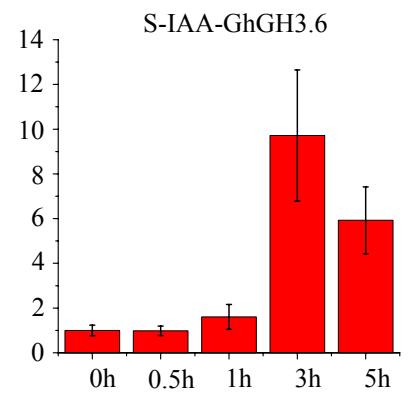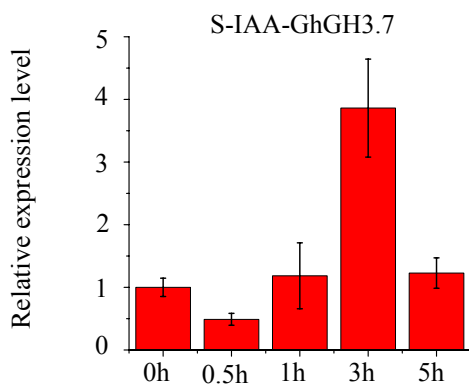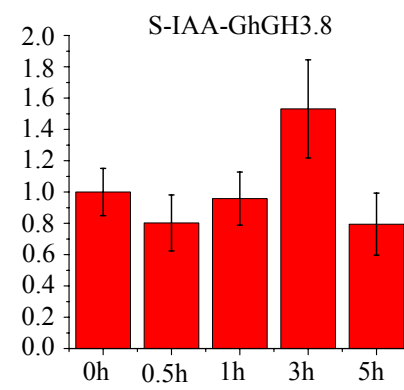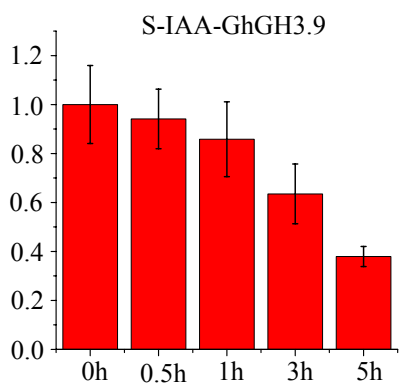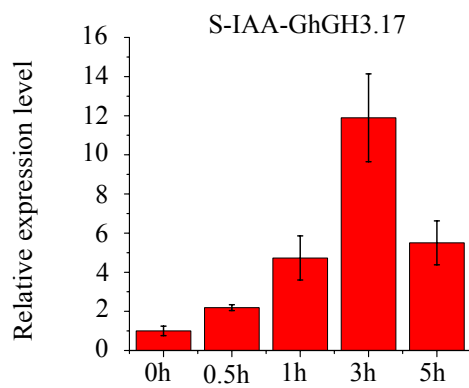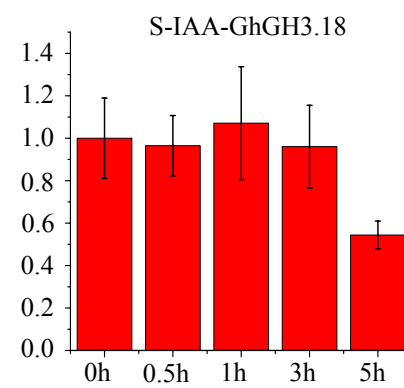

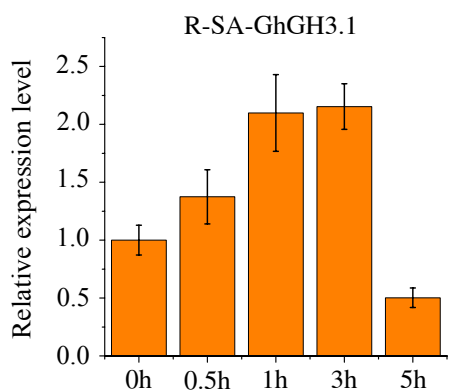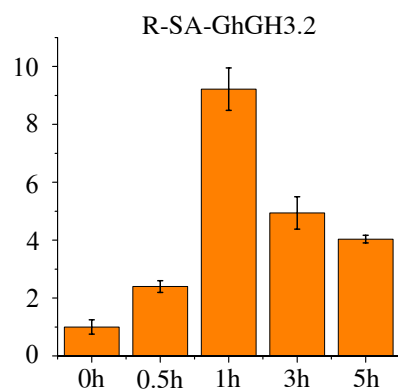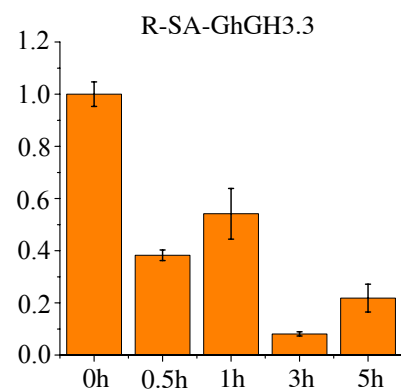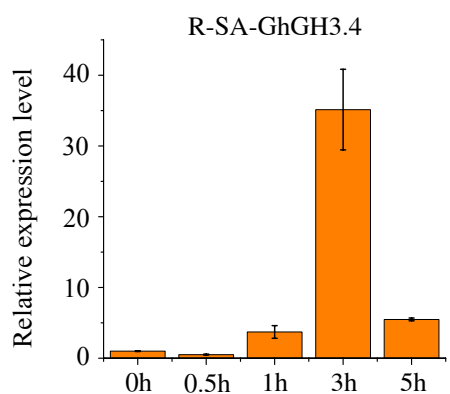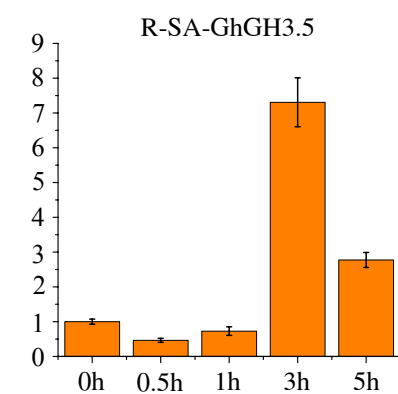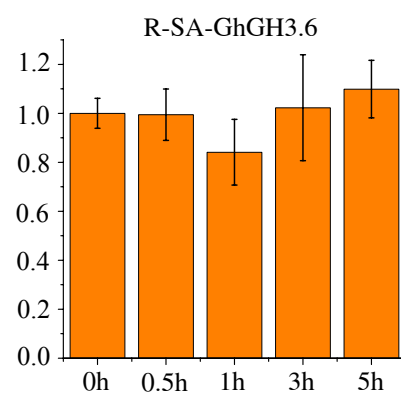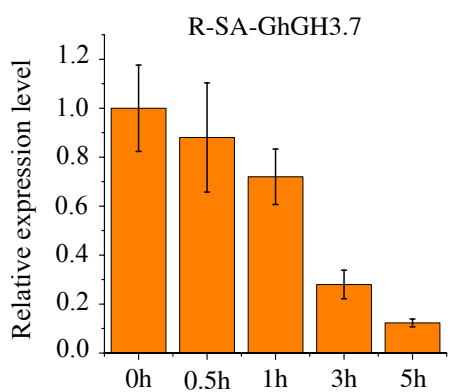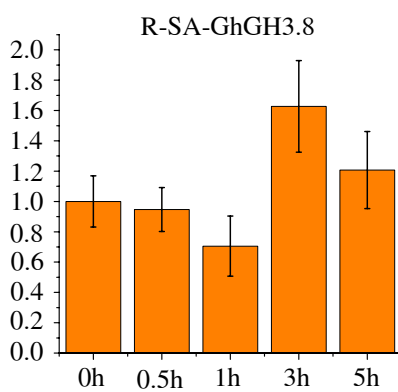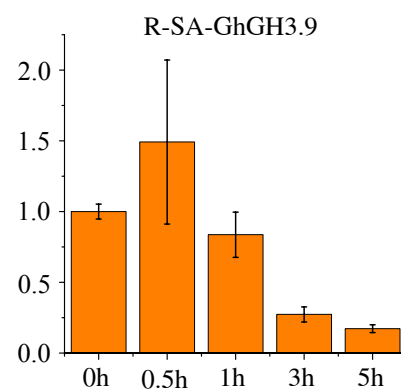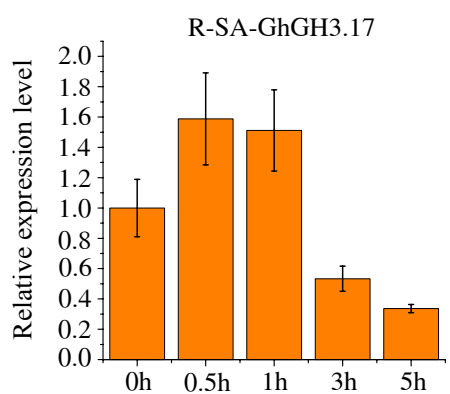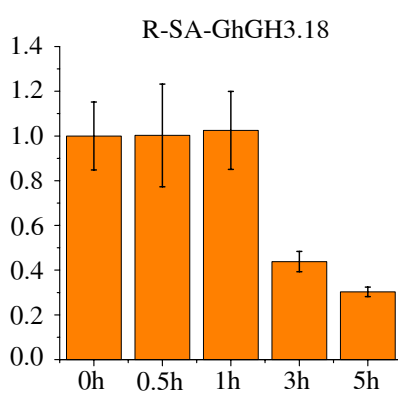

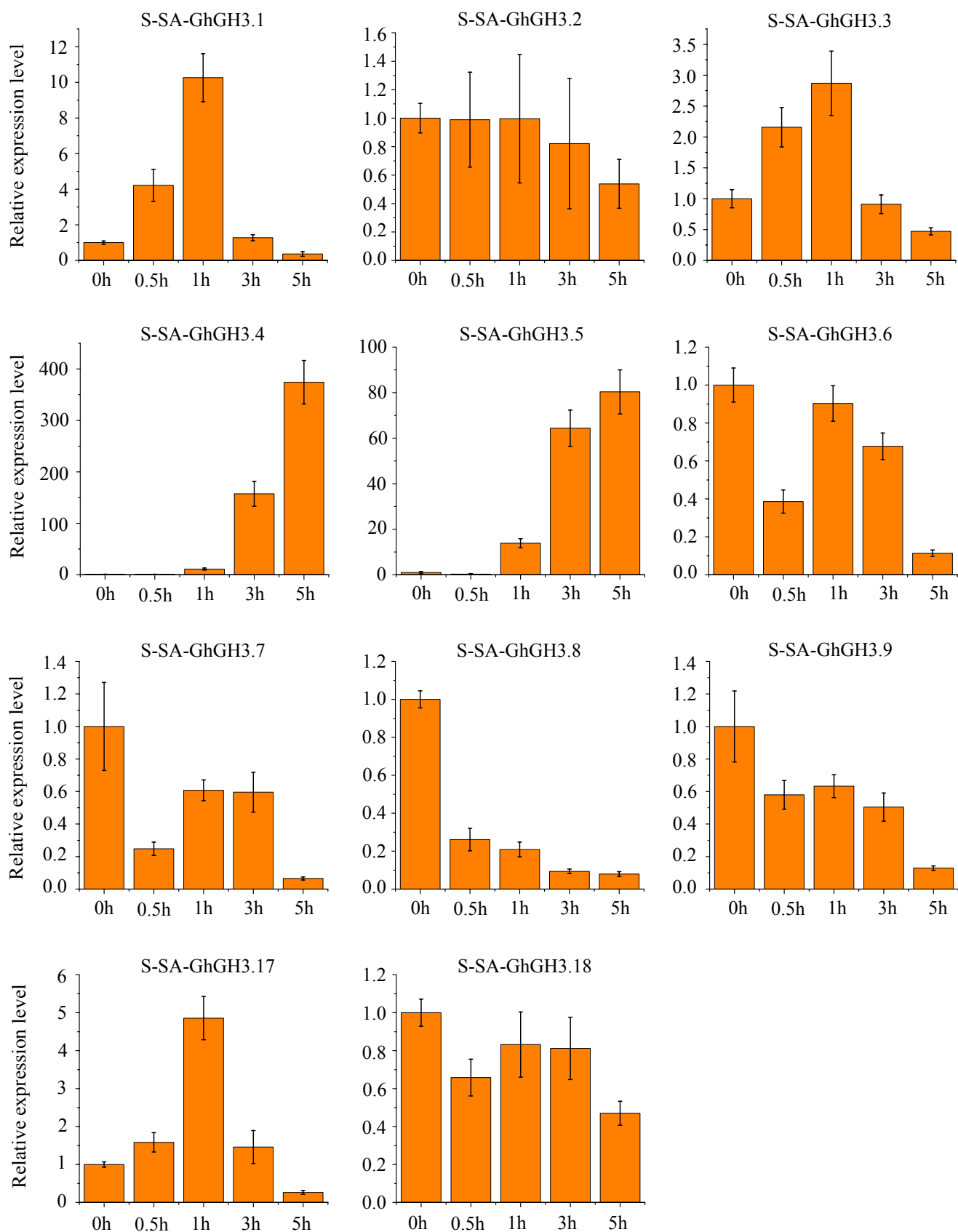

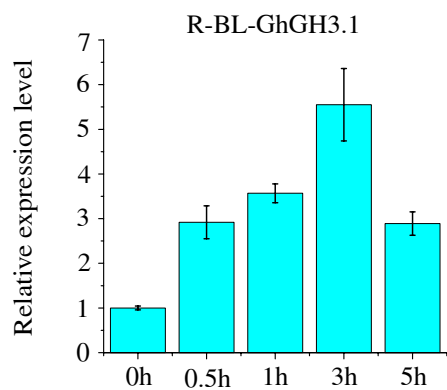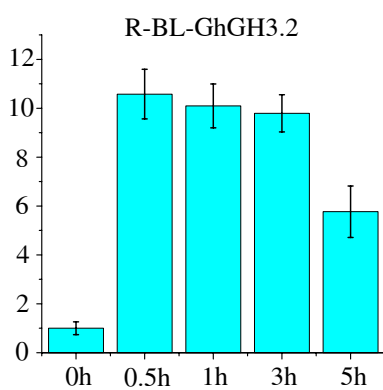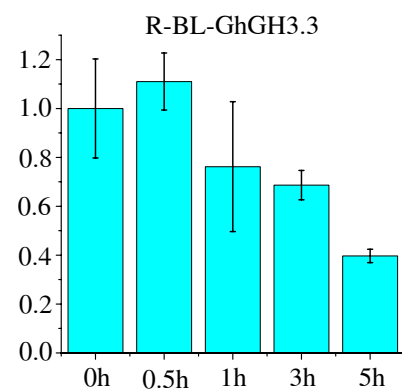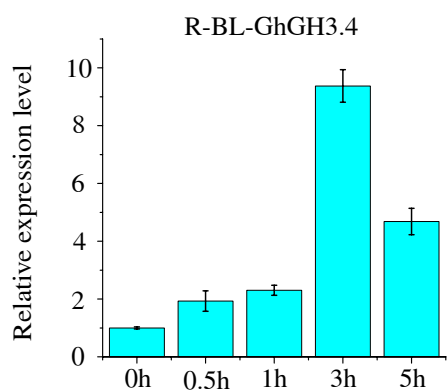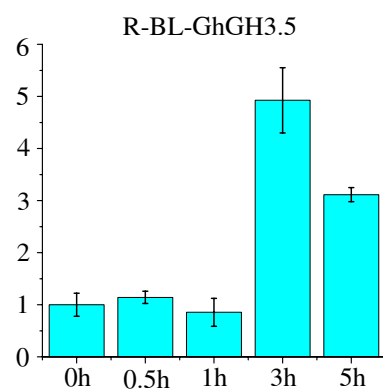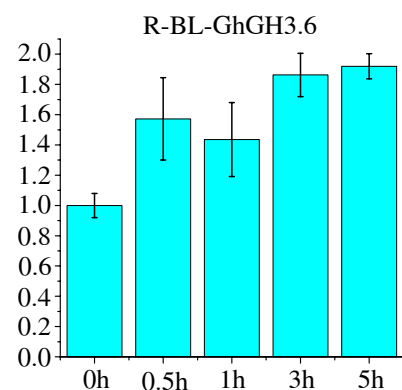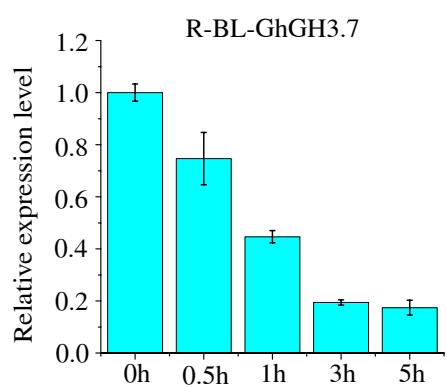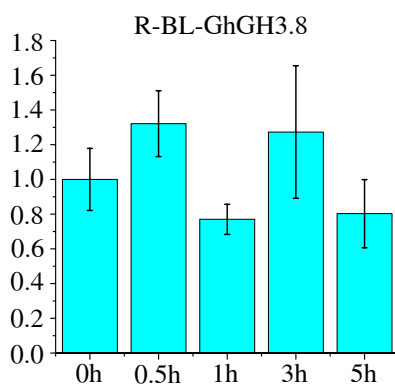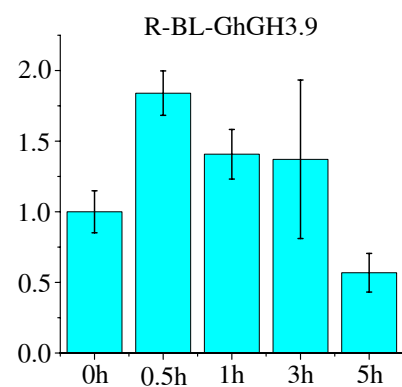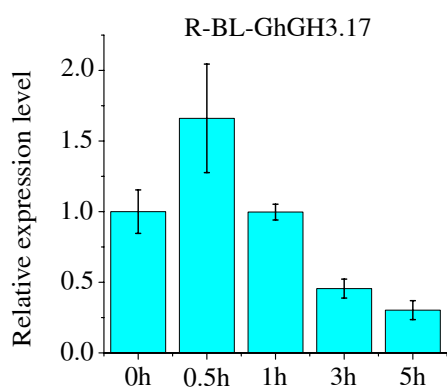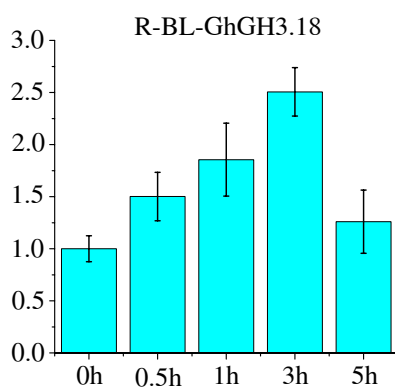

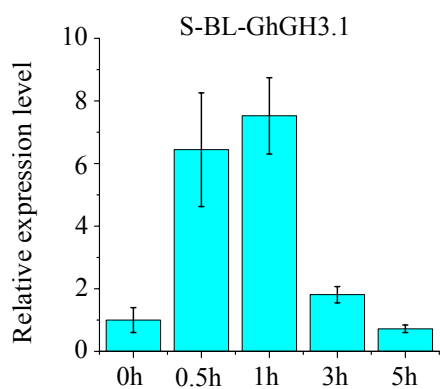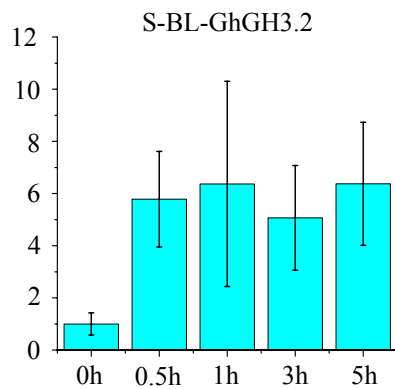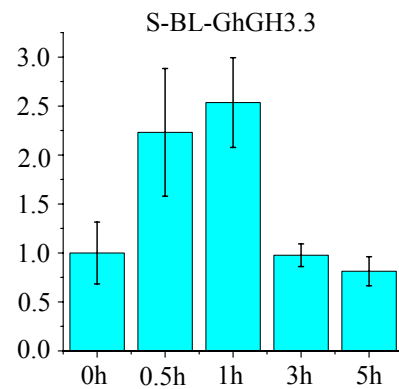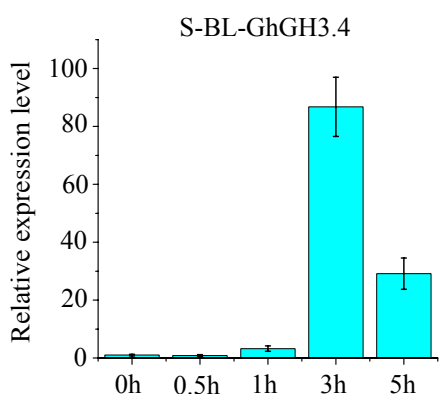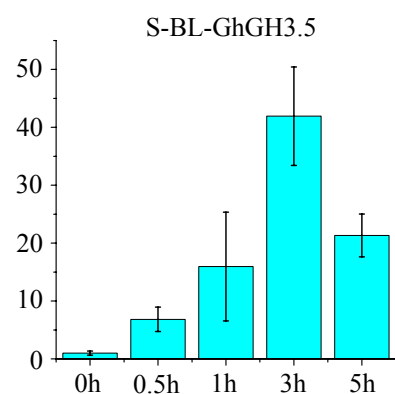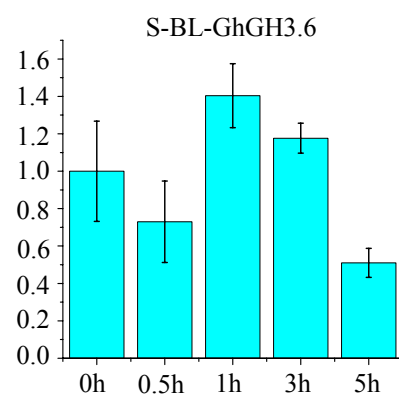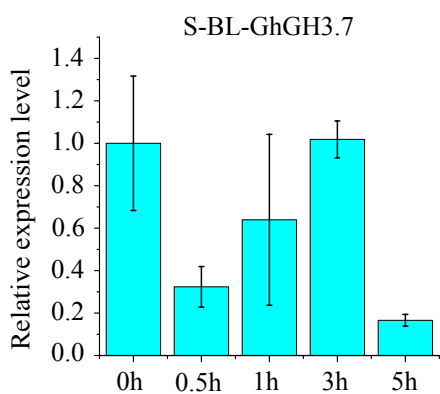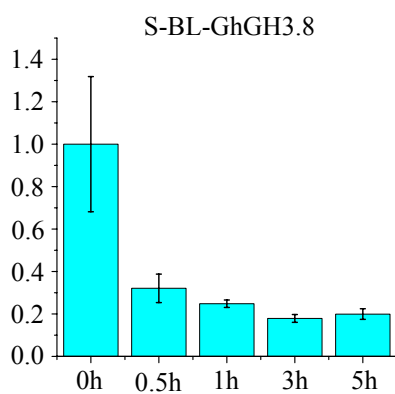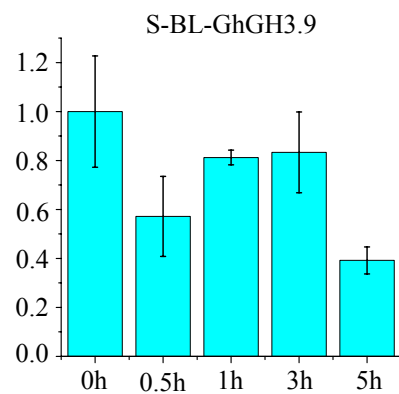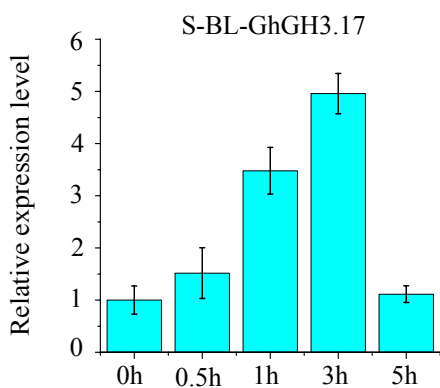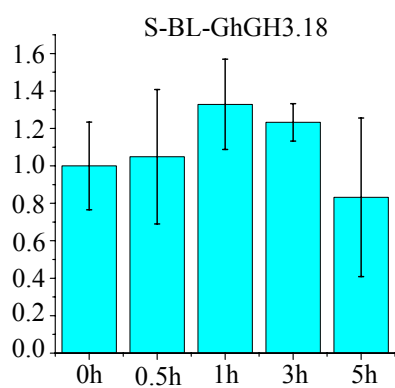

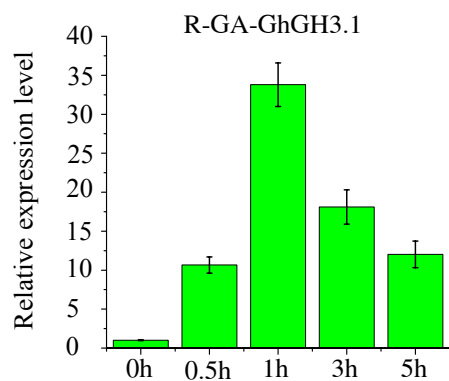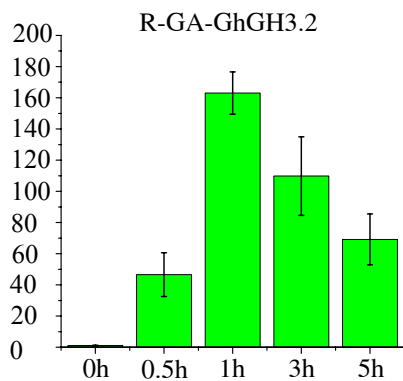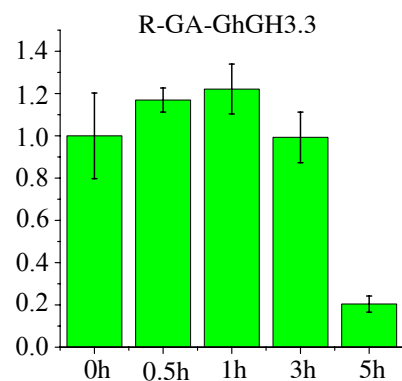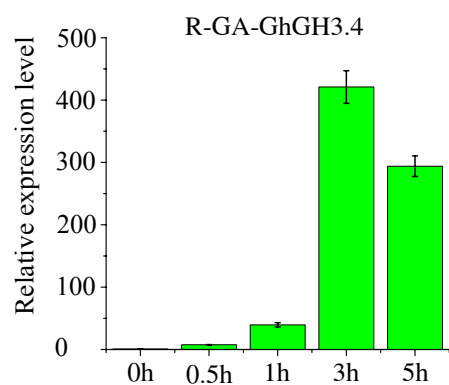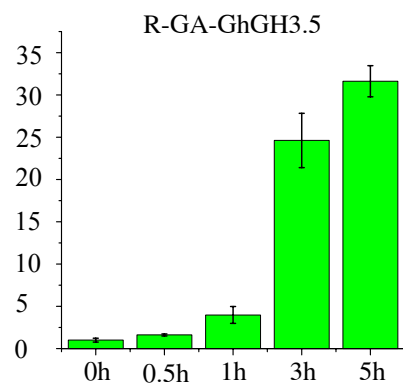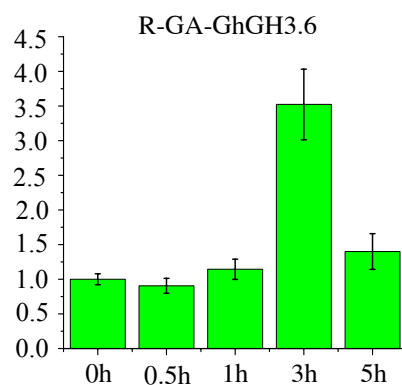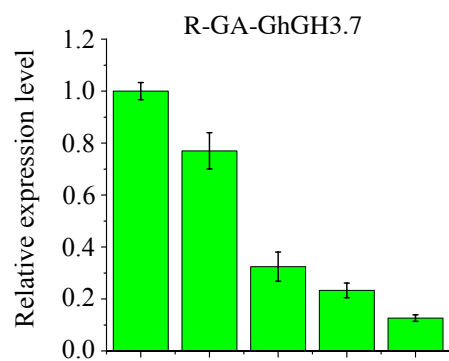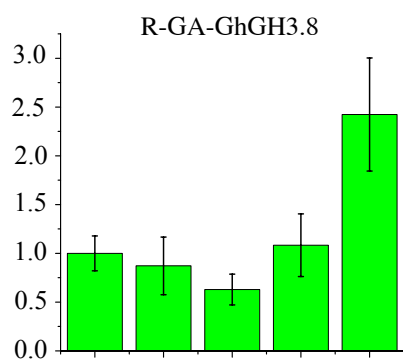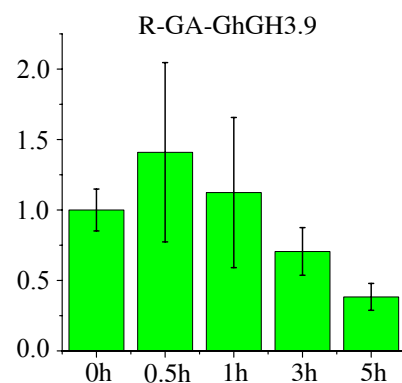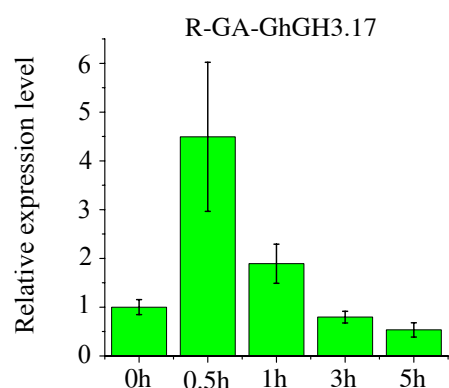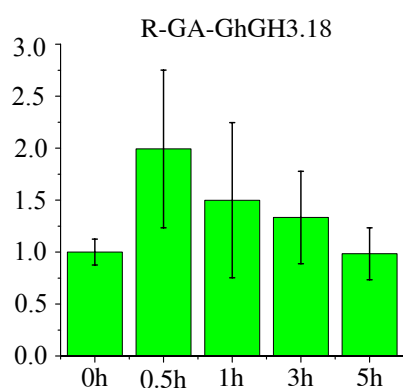

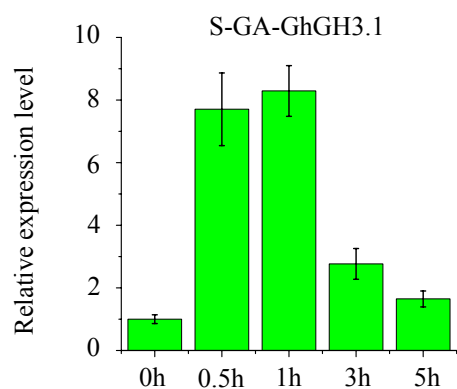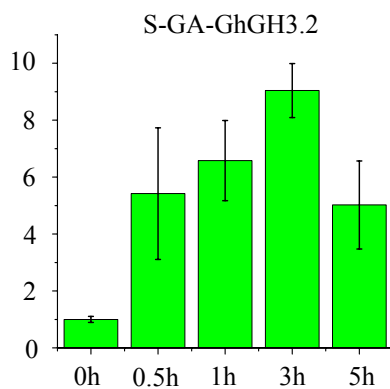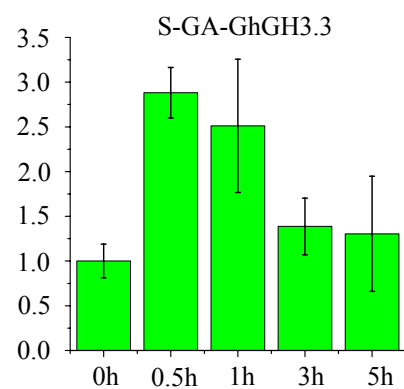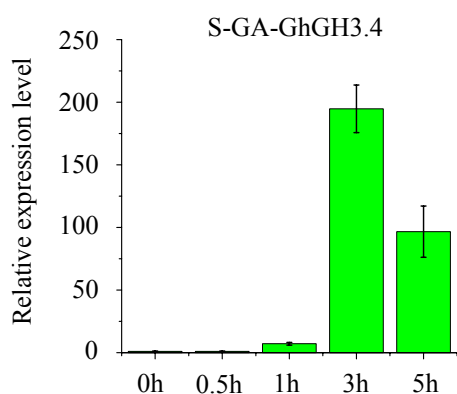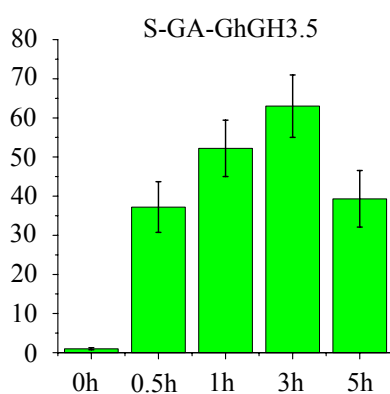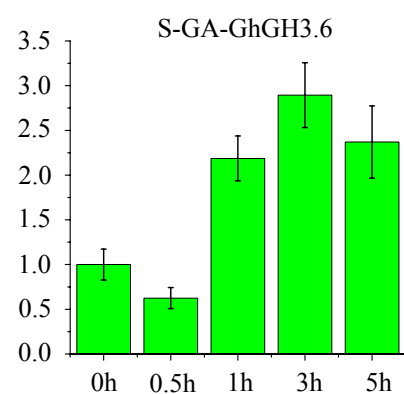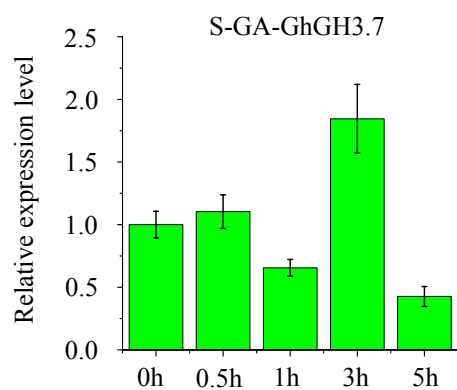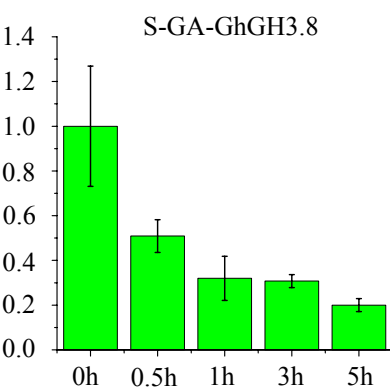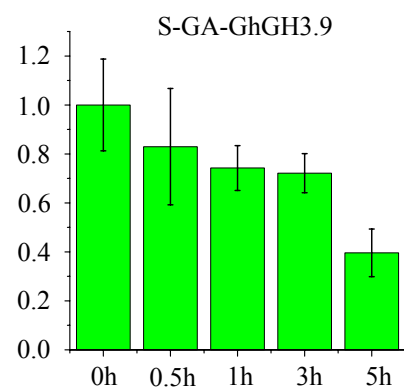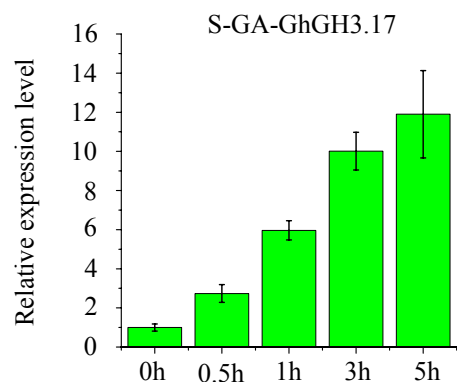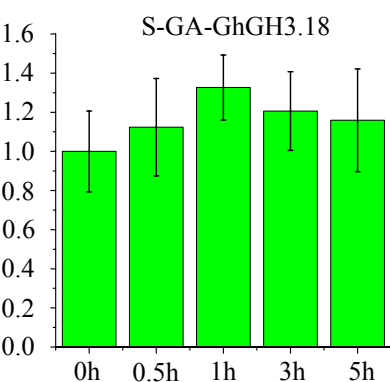

Supplement: Supplementary file 11 — Figure S5. Expression of GhGH3 gene family in response to IAA, SA, BL and GA treatment. R, roots; S, stems. The expression pattern of each GhGH3 gene in response to IAA, SA, BL and GA treatment is also shown. (PDF 1630 kb) [file 12870_2018_1545_MOESM11_ESM.pdf]
