# Supplementary material for: Genome-wide analysis of cotton GH3 subfamily II reveals functional divergence in fiber development, hormone response and plant architecture
Source: BMC Plant Biol. 2018 Dec 12;18:350. doi: 10.1186/s12870-018-1545-5 (PMC6291927; doi:10.1186/s12870-018-1545-5)
Supplement: Supplementary file 3 — Table S2. Characteristics of subfamily II GH3s in G. arboreum and G.raimondii. Detailed characteristics of GH3s in two diploid cottons are shown. Locus_ID and sequence of GrGH3.5 in G. raimondii (BGI, v1.0) was replaced by that of its analogue in G. raimondii (JGI, v2.0) due to the abnormal length possibly caused by improper genomic assembly. (DOCX 24 kb) [file 12870_2018_1545_MOESM3_ESM.docx]

**Additional file 3 Table S2^a^** Characteristics of subfamily Ⅱ *GH3* in *G. arboreum* and *G. raimondii.*

| Gene name | LocusID | ORF | Introns | Chr. | Position | | |  | Deduced polypeptide | | |
| --- | --- | --- | --- | --- | --- | --- | --- | --- | --- | --- | --- |
|  |  |  |  |  | Start | End | Strand |  | Length  (aa) | Wm  (Da) | pI |
| GaGH3.1 | Cotton_A_21697 | 1794 | 2 | chr8 | 39254294 | 39256290 | + |  | 598 | 67.775.51 | 6.41 |
| GaGH3.2 | Cotton_A_24589 | 1791 | 2 | chr6 | 31541886 | 31543863 | + |  | 597 | 67.972.59 | 6.19 |
| GaGH3.3 | Cotton_A_20976 | 1806 | 2 | chr8 | 64011176 | 64013721 | + |  | 602 | 68.039.54 | 5.43 |
| GaGH3.4 | Cotton_A_26931 | 1770 | 2 | chr10 | 45860251 | 45862205 | - |  | 590 | 67.177.11 | 5.83 |
| GaGH3.5 | Cotton_A_26579 | 1818 | 2 | chr4 | 23704018 | 23706037 | - |  | 606 | 68.491.46 | 5.55 |
| GaGH3.6 | Cotton_A_21669 | 1839 | 2 | chr7 | 126647480 | 126649556 | + |  | 613 | 69.484.43 | 5.97 |
| GaGH3.7 | Cotton_A_21671 | 1839 | 2 | chr7 | 126807160 | 126809321 | + |  | 613 | 69.241.17 | 6.08 |
| GaGH3.8 | Cotton_A_08935 | 1845 | 2 | chr5 | 9512417 | 9514465 | + |  | 615 | 69.035.05 | 5.51 |
| GaGH3.9 | Cotton_A_23308 | 1785 | 3 | chr4 | 26984170 | 26987339 | - |  | 595 | 67.017.07 | 5.58 |
| GaGH3.17 | Cotton_A_30234 | 1851 | 3 | chr13 | 21905343 | 21907539 | - |  | 617 | 70.185.47 | 5.73 |
|  |  |  |  |  |  |  |  |  |  |  |  |
| GrGH3.1 | Cotton_D_gene_10016436 | 1794 | 2 | chr10 | 4338979 | 4341532 | - |  | 598 | 67.725.41 | 6.55 |
| GrGH3.2 | Cotton_D_gene_10038257 | 1794 | 2 | chr8 | 66334542 | 66336525 | - |  | 598 | 67.865.49 | 5.97 |
| GrGH3.3 | Cotton_D_gene_10029471 | 1806 | 2 | chr13 | 38763908 | 38766907 | - |  | 602 | 67.995.43 | 5.59 |
| GrGH3.4 | Cotton_D_gene_10032960 | 1794 | 3 | Chr7 | 51464567 | 51472472 | - |  | 598 | 67.649.48 | 5.53 |
| GrGH3.5 | Gorai.007G219500.1 | 1821 | 2 | Chr7 | 25218502 | 25220988 | + |  | 606 | 68.478.51 | 5.53 |
| GrGH3.6 | Cotton_D_gene_10011277 | 1839 | 2 | Chr2 | 42358333 | 42360409 | - |  | 613 | 69.446.48 | 5.91 |
| GrGH3.7 | Cotton_D_gene_10011274 | 1620 | 2 | Chr2 | 42192566 | 42194506 | - |  | 540 | 60.754.56 | 5.79 |
| GrGH3.8 | Cotton_D_gene_10029688 | 1845 | 2 | Chr5 | 38751899 | 38754698 | - |  | 615 | 69.169.19 | 5.32 |
| GrGH3.9 | Cotton_D_gene_10035892 | 1785 | 3 | Chr7 | 45925795 | 45929197 | + |  | 595 | 66.937.73 | 5.36 |
| GrGH3.17 | Cotton_D_gene_10014730 | 1851 | 3 | Chr5 | 36262036 | 36264240 | - |  | 617 | 70.246.60 | 6.28 |

NOTE: Detailed characteristics of *GH3s* in two diploid cottons are shown. Locus_ID and sequence of *GrGH3.5* in *G. raimondii* (BGI, v1.0) was replaced by that of its analogue in *G. raimondii* (JGI, v2.0) due to the abnormal length possibly caused by improper genomic assembly.
