# Supplementary material for: Genome-wide analysis of cotton GH3 subfamily II reveals functional divergence in fiber development, hormone response and plant architecture
Source: BMC Plant Biol. 2018 Dec 12;18:350. doi: 10.1186/s12870-018-1545-5 (PMC6291927; doi:10.1186/s12870-018-1545-5)
Supplement: Supplementary file 4 — Table S3. Comparison of characteristics of GH3s identified in two genome assemblies of G. hirsutum. (DOCX 19 kb) [file 12870_2018_1545_MOESM4_ESM.docx]

**Additional file 4 Table S3** Comparison of characteristics of *GH3s* identified in two genome assemblies of *G. hirsutum.*

| Gene name | NAU-NBI, v1.1 | |  | JGI, v3.1 | | Introns | Chr. | Identities | frame shift |
| --- | --- | --- | --- | --- | --- | --- | --- | --- | --- |
|  | Locus ID | ORF |  | Locus ID | ORF |  |  |  |  |
| Gh_AGH3.1 | Gh_A03G1628 | 1,797 |  | Gohir.A03G185300.1 | 1797 | 2 | A03 | 99.83 | no |
| Gh_DGH3.1 | Gh_D02G2045 | 1,797 |  | Gohir.D02G206800.1 | 1797 | 2 | D02 | 99.89 | no |
| Gh_AGH3.2 | Gh_A12G0181 | 1,794 |  | Gohir.A12G020800.1 | 1794 | 2 | A12 | 99.94 | no |
| Gh_DGH3.2 | Gh_D12G0182 | 1,797 |  | Gohir.D12G021100.1 | 1797 | 2 | D12 | 100 | no |
| Gh_AGH3.3 | Gh_A13G0392 | 1,809 |  | Gohir.A13G044500.1 | 1809 | 2 | A13 | 99.83 | no |
| Gh_DGH3.3 | Gh_D13G0434 | 1,809 |  | Gohir.D13G043600.1 | 1809 | 2 | D13 | 100 | no |
| Gh_AGH3.4 | Gh_A11G0443 | 1,779 |  | Gohir.A11G047900.1 | 1779 | 2 | A11 | 100 | no |
| Gh_DGH3.4 | Gh_D11G0514 | 1,779 |  | Gohir.D11G051500.1 | 1773 | 2 | D11 | 99.94 | no |
| Gh_AGH3.5 | Gh_A11G1993 | 1,821 |  | Gohir.A11G219100.1 | 1821 | 2 | A11 | 99.73 | no |
| Gh_DGH3.5 | Gh_D11G1989 | 1,821 |  | Gohir.D11G204500.1 | 1821 | 2 | D11 | 99.95 | no |
| Gh_AGH3.6 | Gh_A01G0546 | 1,842 |  | Gohir.A01G071700.1 | 1842 | 2 | A01 | 99.89 | no |
| Gh_DGH3.6 | Gh_D01G0557 | 1,842 |  | Gohir.D01G058600.1 | 1842 | 2 | D01 | 99.67 | no |
| Gh_AGH3.7 | Gh_A01G0547 | 1,842 |  | Gohir.A01G071800.1 | 1842 | 2 | A01 | 99.89 | no |
| Gh_DGH3.7 | Gh_D01G0559 | 1,842 |  | Gohir.D01G058700.1 | 1842 | 2 | D01 | 99.95 | no |
| Gh_AGH3.8 | Gh_A03G1429 | 1,848 |  | Gohir.A03G162400.1 | 1848 | 2 | A03 | 99.84 | no |
| Gh_AGH3.9 | Gh_A11G1054 | 1,788 |  | Gohir.A11G115500.1 | 1788 | 3 | A11 | 100 | no |
| Gh_DGH3.9 | Gh_D11G1209 | 1,788 |  | Gohir.D11G120500.1 | 1788 | 3 | D11 | 100 | no |
| Gh_AGH3.17 | Gh_A03G1354 | 1,935 |  | Gohir.A03G153200.1 | 1935 | 4 | A03 | 100 | no |
| Gh_DGH3.17 | Gh_D02G1794 | 1,935 |  | Gohir.D02G176000.1 | 1935 | 4 | D02 | 99.95 | no |
| Gh_AGH3.18 | Gh_A04G0874 | 1,629 |  | Gohir.A04G102000.1 | 1629 | 4 | A04 | 100 | no |
