# Supplementary material for: Genome-wide analysis of cotton GH3 subfamily II reveals functional divergence in fiber development, hormone response and plant architecture
Source: BMC Plant Biol. 2018 Dec 12;18:350. doi: 10.1186/s12870-018-1545-5 (PMC6291927; doi:10.1186/s12870-018-1545-5)
Supplement: Supplementary file 6 — Figure S3. Multiple sequence alignment of 20 GhGH3s. (PDF 35 kb) [file 12870_2018_1545_MOESM6_ESM.pdf]

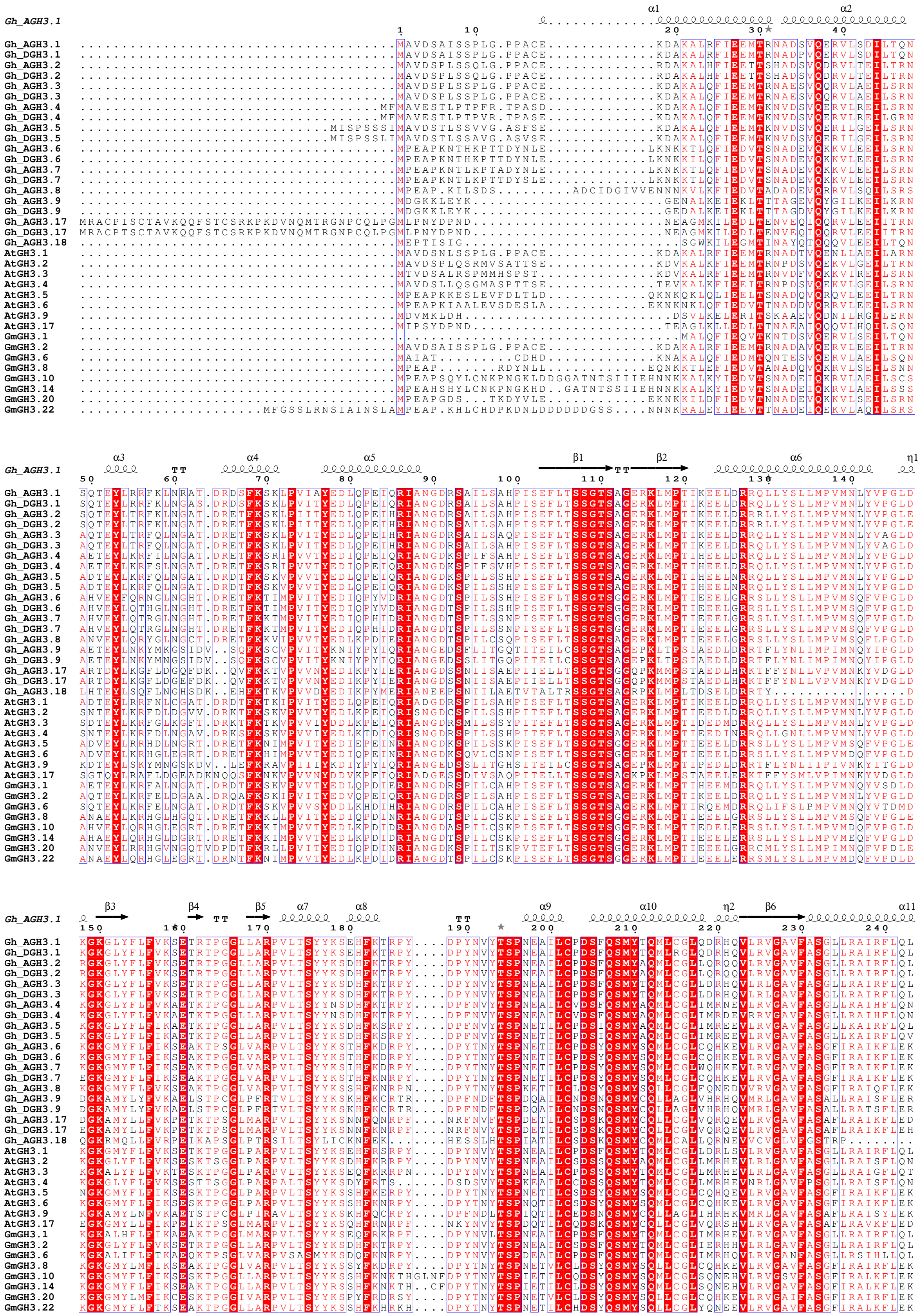
Gh_AGH3.1
α1
α2
α3
α4
α5
β1
β2
α6
η1
β3
β4
β5
α7
α8
α9
α10
η2
β6
α11
Gh_AGH3.1
Gh_DGH3.1
Gh_AGH3.2
Gh_DGH3.2
Gh_AGH3.3
Gh_DGH3.3
Gh_AGH3.4
Gh_DGH3.4
Gh_AGH3.5
Gh_DGH3.5
Gh_AGH3.6
Gh_DGH3.6
Gh_AGH3.7
Gh_DGH3.7
Gh_AGH3.8
Gh_AGH3.9
Gh_DGH3.9
Gh_AGH3.17
Gh_DGH3.17
Gh_AGH3.18
AtGH3.1
AtGH3.2
AtGH3.3
AtGH3.4
AtGH3.5
AtGH3.6
AtGH3.9
AtGH3.17
GmGH3.1
GmGH3.2
GmGH3.6
GmGH3.8
GmGH3.10
GmGH3.14
GmGH3.20
GmGH3.22

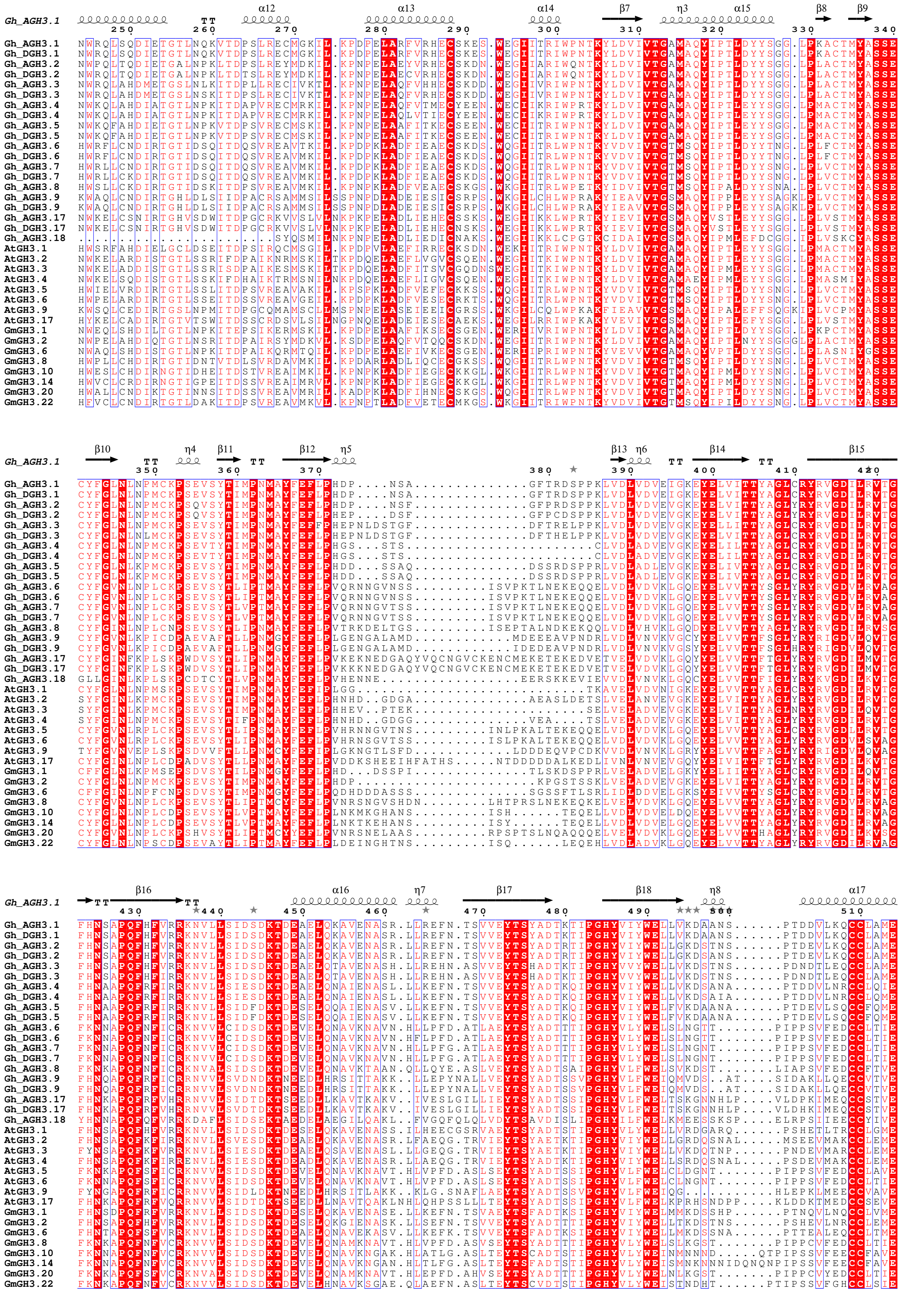
Gh_AGH3.1
α12 α13 α14 β7 η3 α15 β8 β9
β10 η4 β11 β12 η5 β13 η6 β14 β15
β16 α16 η7 β17 β18 η8 α17
Gh_AGH3.1
Gh_DGH3.1
Gh_AGH3.2
Gh_DGH3.2
Gh_AGH3.3
Gh_DGH3.3
Gh_AGH3.4
Gh_DGH3.4
Gh_AGH3.5
Gh_DGH3.5
Gh_AGH3.6
Gh_DGH3.6
Gh_AGH3.7
Gh_DGH3.7
Gh_AGH3.8
Gh_AGH3.9
Gh_DGH3.9
Gh_AGH3.17
Gh_DGH3.17
Gh_AGH3.18
AtGH3.1
AtGH3.2
AtGH3.3
AtGH3.4
AtGH3.5
AtGH3.6
AtGH3.9
AtGH3.17
GmGH3.1
GmGH3.2
GmGH3.6
GmGH3.8
GmGH3.10
GmGH3.14
GmGH3.20
GmGH3.22

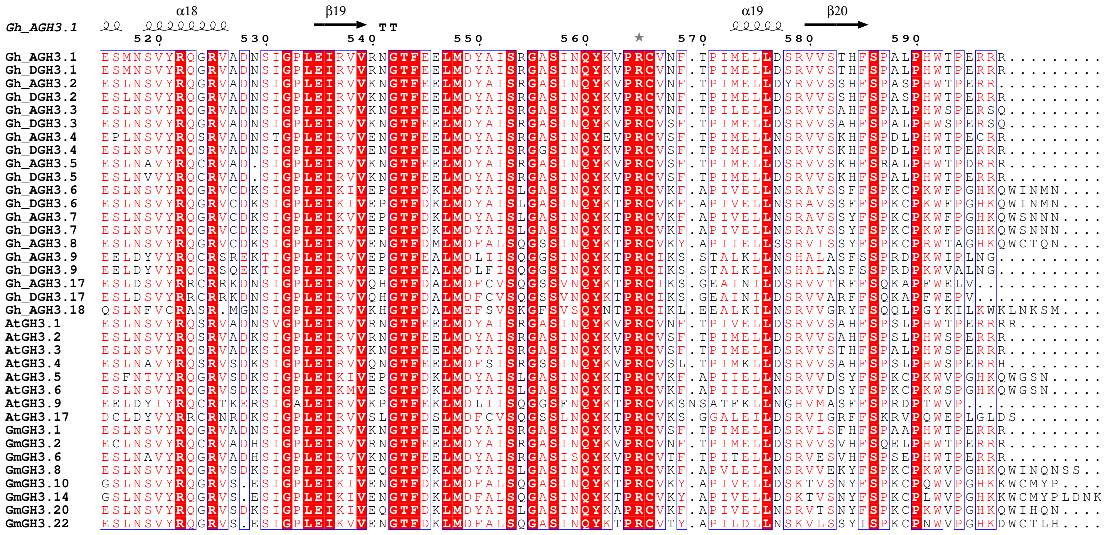
Gh_AGH3.1
α18
β19
TT
α19
β20
520
530
540
550
560
570
580
590
Gh_AGH3.1
Gh_DGH3.1
Gh_AGH3.2
Gh_DGH3.2
Gh_AGH3.3
Gh_DGH3.3
Gh_AGH3.4
Gh_DGH3.4
Gh_AGH3.5
Gh_DGH3.5
Gh_AGH3.6
Gh_DGH3.6
Gh_AGH3.7
Gh_DGH3.7
Gh_AGH3.8
Gh_AGH3.9
Gh_DGH3.9
Gh_AGH3.17
Gh_DGH3.17
Gh_AGH3.18
AtGH3.1
AtGH3.2
AtGH3.3
AtGH3.4
AtGH3.5
AtGH3.6
AtGH3.9
AtGH3.17
GmGH3.1
GmGH3.2
GmGH3.6
GmGH3.8
GmGH3.10
GmGH3.14
GmGH3.20
GmGH3.22
