# Supplementary material for: Genome-wide analysis of cotton GH3 subfamily II reveals functional divergence in fiber development, hormone response and plant architecture
Source: BMC Plant Biol. 2018 Dec 12;18:350. doi: 10.1186/s12870-018-1545-5 (PMC6291927; doi:10.1186/s12870-018-1545-5)
Supplement: Supplementary file 8 — Table S4. Primers used in this study. (DOCX 23 kb) [file 12870_2018_1545_MOESM8_ESM.docx]

**Additional file 8 Table S4** Primers used in this study

| **name** | **Sequence (5'->3')** | **Primers**  **Length** | **Primers location** | | **Tm** | **GC%** | **complementarity** | | **Product length(bp)** |
| --- | --- | --- | --- | --- | --- | --- | --- | --- | --- |
|  |  |  | **Start** | **Stop** |  |  | **Self** | **Self 3'** |  |
| ***GhGH3.1*** |  |  |  |  |  |  |  |  | **143** |
|  | **F: CGTTTACACCAGCCCCAACG** | **20** | **573** | **592** | **61.85** | **60.00** | **4.00** | **2.00** |  |
|  | **R: TGATTGCACGGAGGAGACCG** | **20** | **715** | **696** | **62.23** | **60.00** | **4.00** | **2.00** |  |
|  |  |  |  |  |  |  |  |  |  |
| ***GhGH3.2*** |  |  |  |  |  |  |  |  | **117** |
|  | **F: GCAGTGTTCGCCTCAGGTTT** | **20** | **682** | **701** | **61.17** | **55.00** | **5.00** | **0.00** |  |
|  | **R: TAGTGAAGGGTCGGTGAGTTTAGG** | **24** | **798** | **775** | **61.59** | **50.00** | **2.00** | **0.00** |  |
|  |  |  |  |  |  |  |  |  |  |
| ***GhGH3.3*** |  |  |  |  |  |  |  |  | **100** |
|  | **F: CGTGGATGTGGAAGTTGGGAAA** | **22** | **1182** | **1203** | **61.07** | **50.00** | **3.00** | **0.00** |  |
|  | **R: GAAACCGGTGACGCGAAGAA** | **20** | **1281** | **1262** | **61.22** | **55.00** | **6.00** | **0.00** |  |
|  |  |  |  |  |  |  |  |  |  |
| ***GhGH3.4*** |  |  |  |  |  |  |  |  | **117** |
|  | **F: CGAACTCCGGCTTCCGATAAA** | **21** | **40** | **60** | **60.47** | **52.38** | **4.00** | **0.00** |  |
|  | **R: CTCGGCATTTCGGCTTAGGA** | **20** | **156** | **137** | **60.18** | **55.00** | **3.00** | **0.00** |  |
|  |  |  |  |  |  |  |  |  |  |
| ***GhGH3.5*** |  |  |  |  |  |  |  |  | **119** |
|  | **F: TGATGACTCGTCCGCTCAAGA** | **21** | **1140** | **1160** | **61.22** | **52.38** | **5.00** | **2.00** |  |
|  | **R: GGCACAACCCGGCATAAGTAG** | **21** | **1258** | **1238** | **61.35** | **57.14** | **4.00** | **0.00** |  |
|  |  |  |  |  |  |  |  |  |  |
| ***GhGH3.6*** |  |  |  |  |  |  |  |  | **116** |
|  | **F: GTCCCAAATGGTTTCCCGGC** | **20** | **454** | **473** | **61.88** | **60.00** | **4.00** | **2.00** |  |
|  | **R: GCAAAAACAGTTCTTATGCACTTCC** | **25** | **569** | **545** | **59.82** | **40.00** | **7.00** | **0.00** |  |
|  |  |  |  |  |  |  |  |  |  |
| ***GhGH3.7*** |  |  |  |  |  |  |  |  | **81** |
|  | **F: GGGAAATGAAGGGAGAGATGCT** | **21** | **1009** | **1029** | **61.01** | **52.38** | **4.00** | **0.00** |  |
|  | **R: TCCCAAAGATGATCACACCCACA** | **21** | **1130** | **1110** | **61.14** | **52.38** | **4.00** | **0.00** |  |
|  |  |  |  |  |  |  |  |  |  |
| ***GhGH3.8*** |  |  |  |  |  |  |  |  | **122** |
|  | **F: TGCACCATGTACGCTTCATCC** | **22** | **1** | **22** | **59.83** | **50.00** | **2.00** | **2.00** |  |
|  | **R: GTCACCGGCAAGAACTCGAAA** | **23** | **81** | **59** | **61.85** | **47.83** | **6.00** | **0.00** |  |
|  |  |  |  |  |  |  |  |  |  |
| ***GhGH3.9*** |  |  |  |  |  |  |  |  | **96** |
|  | **F: AGCATCTGTAGCCGTCCATCT** | **21** | **829** | **849** | **61.03** | **52.38** | **2.00** | **0.00** |  |
|  | **R: CTGTGCCATGGAGCCAGTAAC** | **21** | **924** | **904** | **61.28** | **57.14** | **6.00** | **3.00** |  |
|  |  |  |  |  |  |  |  |  |  |
| ***GhGH3.17*** |  |  |  |  |  |  |  |  | **154** |
|  | **F: CGTGCATCGGAGGAATGTCG** | **20** | **1437** | **1456** | **61.48** | **60.00** | **4.00** | **2.00** |  |
|  | **R: TGGTATGGAGGAAGTGTCGGC** | **21** | **1590** | **1570** | **61.84** | **57.14** | **2.00** | **2.00** |  |
|  |  |  |  |  |  |  |  |  |  |
| ***GhGH3.18*** |  |  |  |  |  |  |  |  | **126** |
|  | **F: GCAGGATGGGCAATTCGATAGG** | **22** | **1391** | **1412** | **61.45** | **54.55** | **4.00** | **0.00** |  |
|  | **R: TGCATCGAGGTGTGTTGTATTGG** | **23** | **1516** | **1494** | **61.42** | **47.83** | **4.00** | **0.00** |  |
|  |  |  |  |  |  |  |  |  |  |
| ***GhHIS3*** |  |  |  |  |  |  |  |  | **118** |
|  | **F:** **GACACCAACCTTTGCGCGAT** | **20** | **323** | **342** | **61.85** | **55.00** | **4.00** | **2.00** |  |
|  | **R:** **AGCGACTGATCCACACTTCTG** | **21** | **440** | **420** | **60.07** | **52.38** | **4.00** | **2.00** |  |
|  |  |  |  |  |  |  |  |  |  |
